# Machine learning predicts the short-term requirement for invasive ventilation among Australian critically ill COVID-19 patients

**DOI:** 10.1371/journal.pone.0276509

**Published:** 2022-10-26

**Authors:** Roshan Karri, Yi-Ping Phoebe Chen, Aidan J. C. Burrell, Jahan C. Penny-Dimri, Tessa Broadley, Tony Trapani, Adam M. Deane, Andrew A. Udy, Mark P. Plummer, for the SPRINT-SARI Australia Investigators

**Affiliations:** 1 Royal Melbourne Hospital, Melbourne, Victoria, Australia; 2 Faculty of Science, Technology and Engineering, La Trobe University, Melbourne, Victoria, Australia; 3 Australian and New Zealand Intensive Care Research Centre (ANZIC-RC), School of Public Health and Preventative Medicine, Monash University, Melbourne, Victoria, Australia; 4 Department of Intensive Care and Hyperbaric Medicine, The Alfred Hospital, Melbourne, Victoria, Australia; 5 Intensive Care Unit, Royal Melbourne Hospital, Melbourne, Victoria, Australia; 6 Department of Critical Care, Melbourne Medical School, Melbourne, Victoria, Australia; University of Kurdistan Hewler, IRAQ

## Abstract

**Objective(s):**

To use machine learning (ML) to predict short-term requirements for invasive ventilation in patients with COVID-19 admitted to Australian intensive care units (ICUs).

**Design:**

A machine learning study within a national ICU COVID-19 registry in Australia.

**Participants:**

Adult patients who were spontaneously breathing and admitted to participating ICUs with laboratory-confirmed COVID-19 from 20 February 2020 to 7 March 2021. Patients intubated on day one of their ICU admission were excluded.

**Main outcome measures:**

Six machine learning models predicted the requirement for invasive ventilation by day three of ICU admission from variables recorded on the first calendar day of ICU admission; (1) random forest classifier (RF), (2) decision tree classifier (DT), (3) logistic regression (LR), (4) K neighbours classifier (KNN), (5) support vector machine (SVM), and (6) gradient boosted machine (GBM). Cross-validation was used to assess the area under the receiver operating characteristic curve (AUC), sensitivity, and specificity of machine learning models.

**Results:**

300 ICU admissions collected from 53 ICUs across Australia were included. The median [IQR] age of patients was 59 [50–69] years, 109 (36%) were female and 60 (20%) required invasive ventilation on day two or three. Random forest and Gradient boosted machine were the best performing algorithms, achieving mean (SD) AUCs of 0.69 (0.06) and 0.68 (0.07), and mean sensitivities of 77 (19%) and 81 (17%), respectively.

**Conclusion:**

Machine learning can be used to predict subsequent ventilation in patients with COVID-19 who were spontaneously breathing and admitted to Australian ICUs.

## Introduction

SARS-CoV-2 is a highly transmissible upper respiratory tract virus that causes coronavirus disease 2019 (COVID-19). A striking feature of COVID-19 is rapidly progressive respiratory failure which develops in approximately 5% of infected adults, typically one week after the onset of coryzal symptoms [1, 2]. Globally, two-thirds of adult patients admitted to intensive care with respiratory failure secondary to severe COVID-19 require invasive mechanical ventilation [3]. The institution of mechanical ventilation is strongly associated with poor outcomes in COVID-19—so identifying cohorts at high risk for mechanical ventilation is important to allow therapies to be targeted to specific populations and for resource allocation [4]. Avoiding intubation where possible decreases the risk of the intubation procedure, ventilator-induced lung injury and nosocomial infection. Alternately, delaying an inevitable intubation increases the risk of sudden respiratory arrest and unplanned airway management which exposes staff to a greater risk of infection [5]. Accordingly, developing tools to accurately predict patients at risk of deteriorating is a priority [6].

During the COVID-19 pandemic the prominence of the Electronic Medical Record worldwide has allowed artificial intelligence researchers to interrogate rich databases with machine learning algorithms to improve the speed and accuracy of diagnosis [7, 8], analyse response to therapeutic interventions [9], identify susceptible patients based on genomics [10], and predict mortality [11, 12]. There is a paucity of artificial intelligence research modelling predictors of mechanical ventilation and no studies utilising Australian data. This is important as a limitation of supervised machine learning models is that they are subject to regional bias [13].

The *Short Period Incidence Study of Severe Acute Respiratory Infections* (SPRINT-SARI) Australia registry [4] has been prospectively collecting comprehensive data on critically ill patients with COVID-19 admitted to Australian intensive care units (ICU) from February 2020. The aim of this study was to use the SPRINT-SARI database to develop a machine learning algorithm to predict progression to mechanical ventilation within the first three days of admission to an Australian ICU.

## Methods

This national multicentre inception-cohort study was performed following the recommendations of the STROBE Statement [14]. Ethics approval with full consent waiver was granted under the National Mutual Acceptance scheme by the Alfred Health Human Research Ethics Committee (HREC/16/Alfred/59) or by specific applications at individual sites. Establishment of SPRINT-SARI Australia was approved by the Victorian State Government Chief Health Officer (Professor Brett Sutton) as an Enhanced Surveillance Project "to capture detailed clinical, epidemiological and laboratory data relating to COVID-19 patients in the intensive care setting". The requirement for informed consent was waived as was Site Specific Governance at most contributing sites.

### Study design, setting and participants

The methodology for SPRINT-SARI Australia has been described in detail elsewhere [4]. In brief, the SPRINT-SARI Australia case report form prospectively collected data on all COVID-19 admissions to participating ICUs. Patients had to have a positive polymerase chain reaction (PCR) test for COVID-19 and require ICU admission. Patients without PCR-confirmed COVID-19 and those < 18 years of age were excluded. Data pertaining to baseline demographics, past medical history, clinical characteristics, treatments, and outcomes were collected prospectively and extracted from the SPRINT-SARI Australia database for patients admitted from 20 February 2020 until 7 March 2021. Consistent with previous machine learning studies in severe COVID-19, our study aimed to predict progression to mechanical ventilation within 72 hours of admission using data from the first calendar of admission [13]. Intubation on the first calendar day of ICU admission was thought to reflect pre-ICU variables such that this time window was excluded.

### Variable selection

All available variables were analysed for inclusion in the predictive modelling. Initial exploration of the data involved univariate analysis of variables using Pearson’s Chi-Squared test for categorical variables, and Welch two-sample t-tests for continuous variables. All clinically relevant variables were included in machine learning models regardless of univariate significance. Only data available from day one of ICU admission were used as model inputs. Variable reduction/feature selection was trialled on a per-model basis, removing all inputs with a mutual information score of zero. Sensitivity analysis was subsequently performed, comparing the performance of ‘full’ and ‘reduced variable’ models. A complete list of the input variables included in the final models can be found in the results.

### Outcome definition

A binary outcome variable was defined as “1” if patients received invasive ventilation by either day two or day three of their ICU admission, and “0” if this did not occur [13]. Notably, patients classified as “0” may have ultimately required invasive ventilation at a latter point than day three of their ICU admission. Patients discharged from ICU prior to day three were assigned “0”. Deaths within the designated time-frame were included in the final analysis.

### Data pre-processing

Continuous variables were rescaled to between 0 and 1 using a min-max approach retaining the shape of the continuous distribution. However, rescaling was not used for tree-based approaches, namely random forest, gradient boosting, or decision tree algorithms. For any missing values (see Appendix C of the S1 File) in the final data frame, k nearest neighbour imputation was performed using R statistical software (version 3.5.3) with k = 5. Based on the study protocol and circumstances surrounding data collection, observations missing at random was deemed to be a fair assumption in the context of this investigation [15, 16].

### Machine learning models

Six commonly used [17] classes of machine learning algorithms were explored: (1) random forest classifier, (2) decision tree classifier, (3) logistic regression, (4) K neighbours classifier, (5) support vector machine, and (6) gradient boosted machine [18].

Hyperparameter optimisation was achieved with grid search. Models were supplied the same input variables, and the AUC was the main optimisation metric. Final hyper-parameter values and training metrics are detailed in Appendix A of the S1 File. Machine learning models were constructed using open-source software libraries (Python version 3.6, scikit-learn version 0.24).

### Training and evaluation

Five-fold cross-validation repeated four times was used to assess model performance. Metrics measuring performance were the AUC, sensitivity, and specificity; these were calculated using Youden’s Index at a per-fold basis To account for class imbalance in the data set, minority class oversampling was applied to the training data using SMOTE [19].

### Explanatory model generation

Model accuracy is often achieved through increased complexity, often incurring the cost of compromising explicability. Explanatory modelling of the most performant algorithms in this investigation was achieved with Shapley additive values [20], which provides a unified framework for interpreting feature importance in the context of black-box algorithms. Explanatory modelling was developed for predictions from a test set (20%), from the algorithm trained on a training set (80%). Explanations for correctly classified samples were visualised with a summary plot, where the points on the plot are the change in model output, derived from the Shapley value of that feature, for each patient in the test set [20].

## Results

The raw dataset consisted of 608 patients, of whom 387 (63.7%) were not ventilated on day one of admission. A further 87 patients had inadequate data collected on day one of admission (nil bloods data for the given patient at the relevant site and time-point) and were excluded leaving 300 patients from 53 ICUs included in the final analysis. This included 60 (20%) patients who required invasive ventilation by day three of their ICU admission, and 240 (80%) patients who did not. Median (IQR) age for the final dataset was 59 (50–69) years, comprising of 191 (63.7%) male patients. Inputs utilised in the modelling are shown in Tables 1 (discrete variables) and 2 (continuous variables), along with their population characteristics (stratified by whether or not invasive ventilation was required by day 3) and respective p-values. Variable reduction did not yield a statistically significant improvement in model performance (see Appendix D of the S1 File). A further 26 patients from the original cohort went on to require invasive ventilation beyond day three. The median time to invasive ventilation was two days (Range 2–14, IQR 2–4).

**Table 1:** Discrete input variables’ population characteristics and corresponding p-value (Pearson’s Chi-Squared Test).

Input variable	Received invasive ventilation on day 2 or 3 (%)	Did not receive invasive ventilation on day 2 or 3 (%)	p.value
History of travel to area with documented COVID cases	19 (32)	65 (27)	0.58
Close contact with suspected or confirmed COVID-19 case	42 (70)	142 (59)	0.16
Presence in healthcare facility with documented COVID-19	7 (12)	31 (13)	0.97
Presence in laboratory handling COVID-19 samples	2 (3)	4 (2)	0.76
Arab	7 (12)	14 (6)	0.19
Black	4 (7)	6 (3)	0.23
East Asian	3 (5)	19 (8)	0.62
South Asian	7 (12)	17 (7)	0.37
West Asian	0 (0)	2 (1)	1.00
Latin American	2 (3)	2 (1)	0.38
Caucasian	27 (45)	103 (43)	0.88
Aboriginal/First Nations	1 (2)	0 (0)	0.45
Other Ethnicity	3 (5)	24 (10)	0.34
Unknown Ethnicity	5 (8)	50 (21)	0.04
Male sex at birth	40 (67)	151 (63)	0.70
Female sex at birth	20 (33)	89 (37)	
Transfer from other health facility	3 (5)	20 (8)	0.55
History of fever	47 (78)	188 (78)	1.00
Cough	43 (72)	174 (73)	1.00
Cough with sputum production	15 (25)	67 (28)	0.77
Haemoptysis	0 (0)	10 (4)	0.23
Sore throat	13 (22)	45 (19)	0.74
Rhinorrhoea	4 (7)	29 (12)	0.33
Ear pain	0 (0)	1 (0)	1.00
Wheeze	1 (2)	23 (10)	0.08
Chest pain	10 (17)	52 (22)	0.50
Myalgia	27 (45)	79 (33)	0.11
Joint pain	6 (10)	14 (6)	0.39
Fatigue	38 (63)	147 (61)	0.88
Dyspnoea	46 (77)	177 (74)	0.77
Lower chest wall indrawing	2 (3)	1 (0)	0.19
Headache	15 (25)	44 (18)	0.33
Altered conscious state	4 (7)	23 (10)	0.65
Seizures	0 (0)	0 (0)	0.00
Abdominal pain	3 (5)	19 (8)	0.62
Vomiting/Nausea	16 (27)	64 (27)	1.00
Diarrhoea	24 (40)	63 (26)	0.05
Conjunctivitis	1 (2)	0 (0)	0.45
Skin rash	1 (2)	3 (1)	1.00
Skin ulcers	2 (3)	1 (0)	0.19
Lymphadenopathy	1 (2)	1 (0)	0.86
Bleeding(haemorrhage)	0 (0)	2 (1)	1.00
Bleeding in more than one place	0 (0)	1 (0)	1.00
Loss of smell/taste	10 (1)	27 (11)	0.36
Rigors or sweating	15 (25)	46 (19)	0.41
Severe dehydration	3 (5)	10 (4)	1.00
Type of oxygen saturation reading closest to pre-intubation on day one:			0.02
On O2 therapy	40 (67)	116 (48)	
On room air	20 (33)	124 (52)	
Chronic cardiac disease	11 (18)	29 (12)	0.29
Past ACE inhibitor or A2 blocker use	13 (22)	47 (20)	0.86
Obesity	19 (32)	55 (23)	0.22
Chronic pulmonary disease	5 (8)	21 (9)	1.00
Complicated diabetes	3 (5)	11 (5)	1.00
Uncomplicated diabetes	18 (30)	48 (20)	0.13
Asthma	10 (17)	38 (16)	1.00
Chronic Kidney Disease	2 (3)	13 (5)	0.74
Rheumatological disorder	0 (0)	15 (6)	0.10
Moderate or severe liver disease	1 (2)	3 (1)	1.00
Dementia	0 (0)	0 (0)	0.00
Mild liver disease	0 (0)	6 (3)	0.47
Malnutrition	0 (0)	0 (0)	0.00
Chronic neurological disorder	1 (2)	4 (2)	1.00
Malignant neoplasm	1 (2)	9 (4)	0.69
Smoker	7 (12)	23 (10)	0.81
Chronic haematological disease	2 (3)	6 (3)	1.00
AIDS/HIV	0 (0)	3 (1)	0.88
Chronic immunosuppression	2 (3)	21 (9)	0.25
Readmission	0 (0)	3 (1)	0.88
PaO2 sample type—from ABG with worst P:F ratio for the day:	0	0	0.00
Arterial	54 (90)	167 (70)	
Venous	6 (10)	73 (30)	
High flow nasal cannula therapy required	43 (72)	112 (47)	0.00
Non-invasive ventilation (e.g. BIPAP,CPAP) required	3 (5)	17 (7)	0.77
Vasopressor support required	3 (5)	7 (3)	0.69
Prone positioning required	7 (12)	26 (11)	1.00

**Table 2:** Continuous input variables’ population characteristics and corresponding p-value (Welch Two Sample t-test).

Input variable	Received invasive ventilation on day 2 or 3	Did not receive invasive ventilation on day 2 or 3	p.value
	Median (IQR)	Median (IQ)	
Age (years)	64.5 (52.2–70.0)	58.0 (49.0–69.0)	0.17
Time from onset of symptoms to ICU admission (days)	7 (5–10)	8 (5–11)	0.33
Time spent in hospital prior to ICU admission (hours)	17 (3–57)	9 (3–43)	0.32
Temperature H24 (°C)	38.6 (37.8–39.1)	38.1 (37.3–38.8)	0.06
Heart rate H24 (beats per minute)	94 (87–109)	100 (85–111)	0.64
Respiratory rate -highest for day (breaths per minute)	30 (25–36)	30 (24–36)	0.41
Systolic blood pressure L24 (mmHg)	119 (103–143)	113 (10–132)	0.16
Diastolic blood pressure—from same point as SBP L24 (mmHg)	60 (54–73)	63 (57–75)	0.30
Oxygen saturation (%) closest to pre-intubation on day one	91 (89–94)	92 (88–95)	0.74
Estimated height (cm)	170 (160–178)	170 (164–175)	0.79
Estimated weight (kg)	82 (70–100)	85 (71–100)	0.66
FiO2—from ABG with worst P:F ratio for the day	0.45 (0.36–0.55)	0.35 (0.25–0.46)	0.91
SaO2/SpO2—from ABG with worst P:F ratio for the day (%)	92 (89–95)	93 (87–95)	0.08
PaO2—from ABG with worst P:F ratio for the day (mmHg)	65 (57–73)	60 (50–70)	0.02
PaCO2 -from ABG with worst P:F ratio for the day (mmHg)	34 (32–37)	36 (33–39)	0.02
pH from ABG with worst P:F for the day	7.46 (7.44–7.48)	7.44 (7.42–7.46)	0.00
HCO3- from ABG with worst P:F ratio for the day (mmol/L)	24 (23–26)	24 (23–26)	0.72
Base excess from ABG with worst P:F ratio for the day (mmol/L)	0.9 (-0.4–2.4)	0.6 (-1.1–2.3)	0.18
GCS—lowest for the day	15 (14–15)	15 (14–15)	0.84
Systolic BP—lowest for the day (mmHg)	114 (104–132)	110 (100–121)	0.05
Diastolic BP—from same time point as lowest SBP on day 1 (mmHg)	61 (56–71)	64 (56–72)	0.76
Mean arterial pressure—lowest for day (mmHg)	78 (73–92)	81 (70–89)	0.61
Daily urine output (mL)	911 (500–1229)	925 (582–1206)	0.97
Platelet count—worst value for day (x 10^9 /L)	205 (110–236)	208 (176–243)	0.74
Total bilirubin—worst value for day (μmol/L)	11 (7–13)	10 (8–13)	0.58
Lactate—worst value for day (mmol/L)	1.4 (1.1–2.0)	1.5 (1.2–2.0)	0.96
Creatinine—worst value for day (μmol/L)	79 (66–96)	77 (67–92)	0.85
Number of quadrants in which infiltrates are present on CXR	3 (2–3)	2 (2–3)	0.28
Haemoglobin—lowest value for day (g/L)	134 (123–138)	133 (125–140)	0.64
WBC count—lowest for day (x 10^9 /L)	6.99 (5.80–8.73)	7.15 (5.49–8.90)	0.77
WBC count—highest for day (x 10^9 /L)	8.03 (6.37–9.96)	8.15 (6.32–9.68)	0.97
Lymphocyte count—lowest for day (x 10^9 /L)	0.71 (0.60–0.93)	0.80 (0.66–0.97)	0.13
Neutrophil count—lowest for day (x 10^9 /L)	5.60 (4.22–7.05)	5.17 (3.80–6.59)	0.21
Haematocrit—worst value for day (L/L)	0.39 (0.36–0.41)	0.39 (0.37–0.41)	0.50
APTT/APTR—worst value for day (s)	32 (31–35)	33 (30–37)	0.35
PT—worst value for day (s)	15 (14–19)	15 (14–24)	0.29
INR—worst value for day	1.1 (1.0–1.1)	1.1 (1.0–1.2)	0.02
ALT/SGPT—worst value for day (U/L)	42 (31–56)	47 (34–67)	0.76
AST/SGOT—worst value for day (U/L)	53 (45–58)	54(45–66)	0.97
Glucose—highest for day (mmol/L)	8.8 (7.4–10.4)	9.3 (7.8–11.0)	0.09
Blood Urea Nitrogen—worst value for day (mmol/L)	5.7 (4.9–7.5)	5.9 (4.8–7.5)	0.98
Sodium—worst value for day (mmol/L)	136 (133–137)	136 (134–137)	0.03
Potassium—worst value for day (mmol/L)	3.9 (3.7–4.0)	4.0 (3.8–4.2)	0.01
CRP—worst value for day (mg/L)	107 (84–134)	104 (83–124)	0.07
Daily fluid balance (mL)	8.48 (-278.40–392.08)	126.00 (-253.35–530.60)	0.27

H24—Highest in first 24 hours of hospital admission, L24—Lowest in first 24 hours of hospital admission

‘Day’ refers to the first 24 hours of ICU admission unless otherwise specified.

’Worst’ refers to the worst value as relating to the APACHE II score.

P:F ratio = PaO2 divided by FiO2

### Training and model fit

Final performance metrics (see Appendix A of the S1 File) suggest that despite optimized hyperparameter tuning, all models evaluated in this study suffered from a degree of overfitting.

### Predicting the need for invasive ventilation by day 2 or 3 of ICU admission

The best overall performing machine learning algorithms were gradient boosted machine and random forest classifier, with mean (SD) AUC of 0.68 (0.07) and 0.69 (0.06) respectively. These models additionally demonstrated high mean (SD) sensitivities of 0.81 (0.17) and 0.77 (0.19) respectively. DeLong’s test revealed that there was no significant difference in the performance of gradient boosted machine and random forest classifier (Z = 0.82, p-value = 0.41), and that these both significantly outperformed each of the remaining machine learning algorithms tested. A comprehensive list of DeLong’s test coefficients can be found in Appendix B of the S1 File.

Second in overall performance was support vector machine, with a mean (SD) AUC of 0.65 (0.08), followed by LR with a mean (SD) AUC of 0.64 (0.08). Decision tree was the poorest performing model tested with a mean (SD) AUC of 0.54 (0.07), representing zero class separation capability. A complete outline of the models tested, their AUCs, and additional performance metrics can be seen in Table 3.

**Table 3:** Performance of ML algorithms.

Classifier	AUC	Sensitivity	Specificity
KNN	0.59 +/- 0.07	0.78 +/- 0.24	0.49 +/- 0.20
DT	0.54 +/- 0.07	0.31 +/- 0.13	0.78 +/- 0.05
SVM	0.65 +/- 0.08	0.78 +/- 0.16	0.59 +/- 0.15
GBM	0.68 +/- 0.07	0.81 +/- 0.17	0.58 +/- 0.17
LR	0.64 +/- 0.08	0.75 +/- 0.19	0.59 +/- 0.21
RF	0.69 +/- 0.06	0.77 +/- 0.19	0.62 +/- 0.17

### Explanatory modelling

**Fig 1:**
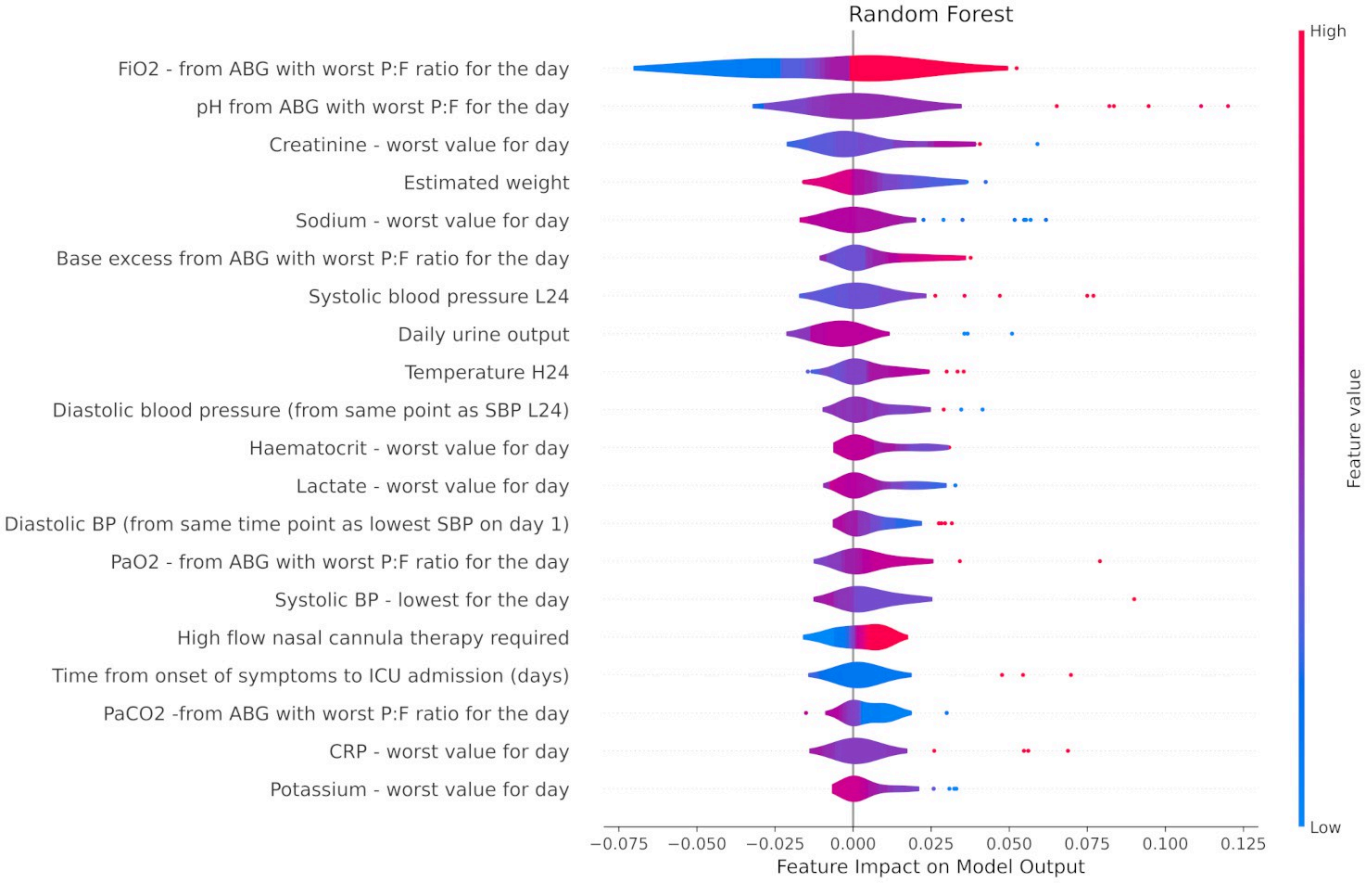
Summary plot showing the 20 most predictive features as defined by the RF model for correctly classified samples. H24—Highest in first 24 hours of hospital admission, L24—Lowest in first 24 hours of hospital admission. ‘Day’ refers to the first 24 hours of ICU admission unless otherwise specified. ’Worst’ refers to the worst value as relating to the APACHE II score. P:F ratio = PaO2 divided by FiO2.

**Fig 2:**
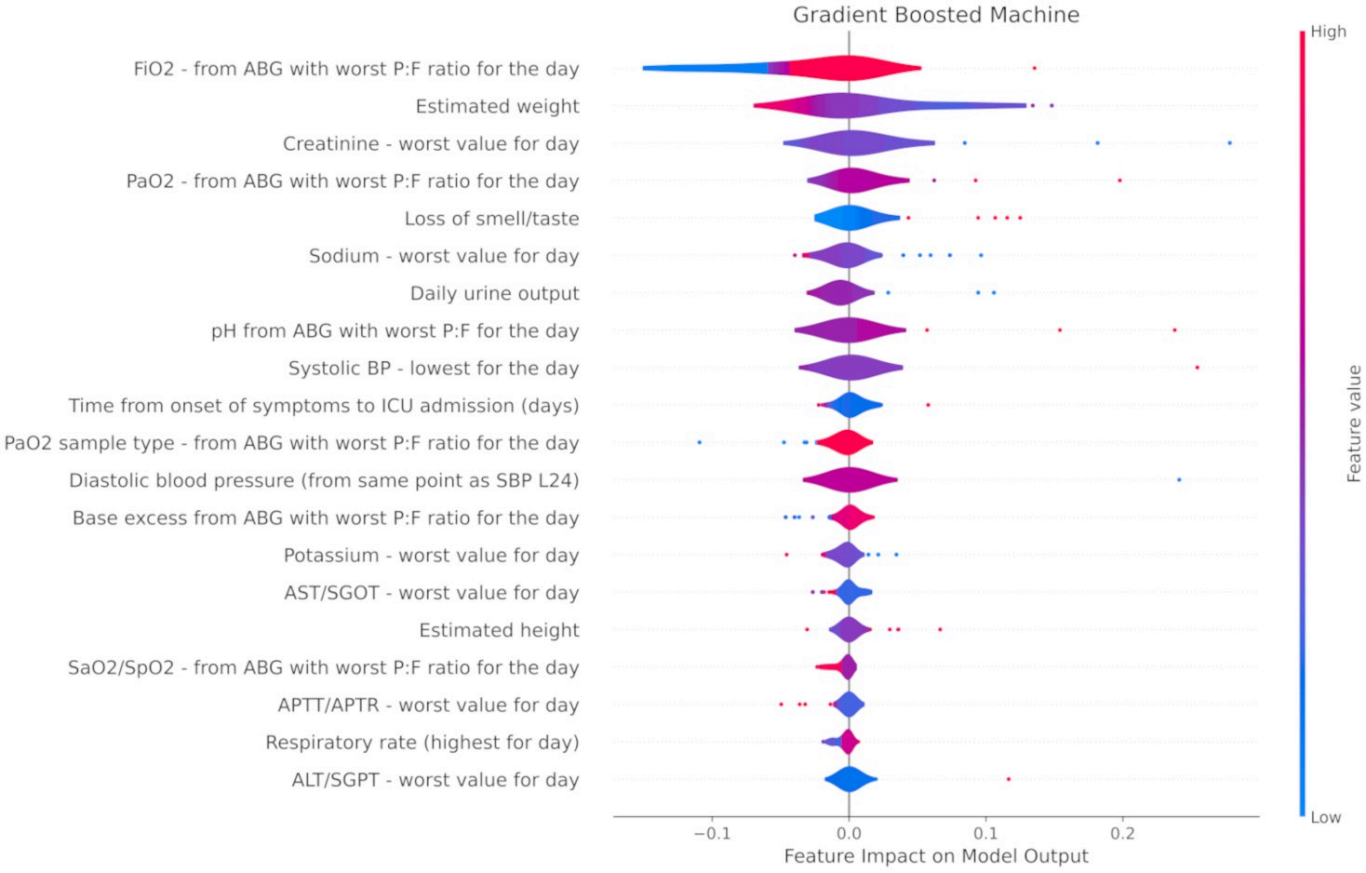
Summary plot showing the 20 most predictive features as defined by the GBM model for correctly classified samples. H24—Highest in first 24 hours of hospital admission, L24—Lowest in first 24 hours of hospital admission. ‘Day’ refers to the first 24 hours of ICU admission unless otherwise specified. ’Worst’ refers to the worst value as relating to the APACHE II score. P:F ratio = PaO2 divided by FiO2.

The top 20 most impactful features that contributed to correct sample classification in RF and GBM are seen in Figs 1 and 2, respectively. Blue and red are indicative of higher and lower variable values respectively, whilst left of the X-axis meridian implies favouring a requirement for short term ventilation. For example, in the case of GBM (Fig 1), the estimated weight variable is red left of the Y-axis, and blue to its right. This broadly suggests that the model attributed a higher risk of short term ventilation to overweight patients. Numerous highly weighted features were shared between the two algorithms, with an apparent focus on arterial blood gas derived data including the fraction of inspired oxygen (FiO_2_), arterial partial pressure of oxygen (PaO_2_), pH, and base excess. Other laboratory derived data (worst plasma sodium, potassium, and lactate levels) and clinical observations (lowest systolic blood pressure and diastolic blood pressure) were also shared between the two models. Minor differences included that gradient boosted machine utilised pulse oximetry derived arterial oxygen saturation (SaO_2_) whereas random forest classifier did not, and, conversely, random forest classifier gave relative importance to arterial partial pressure of carbon dioxide (PaCO_2_) whilst gradient boosted machine did not.

**Fig 3:**
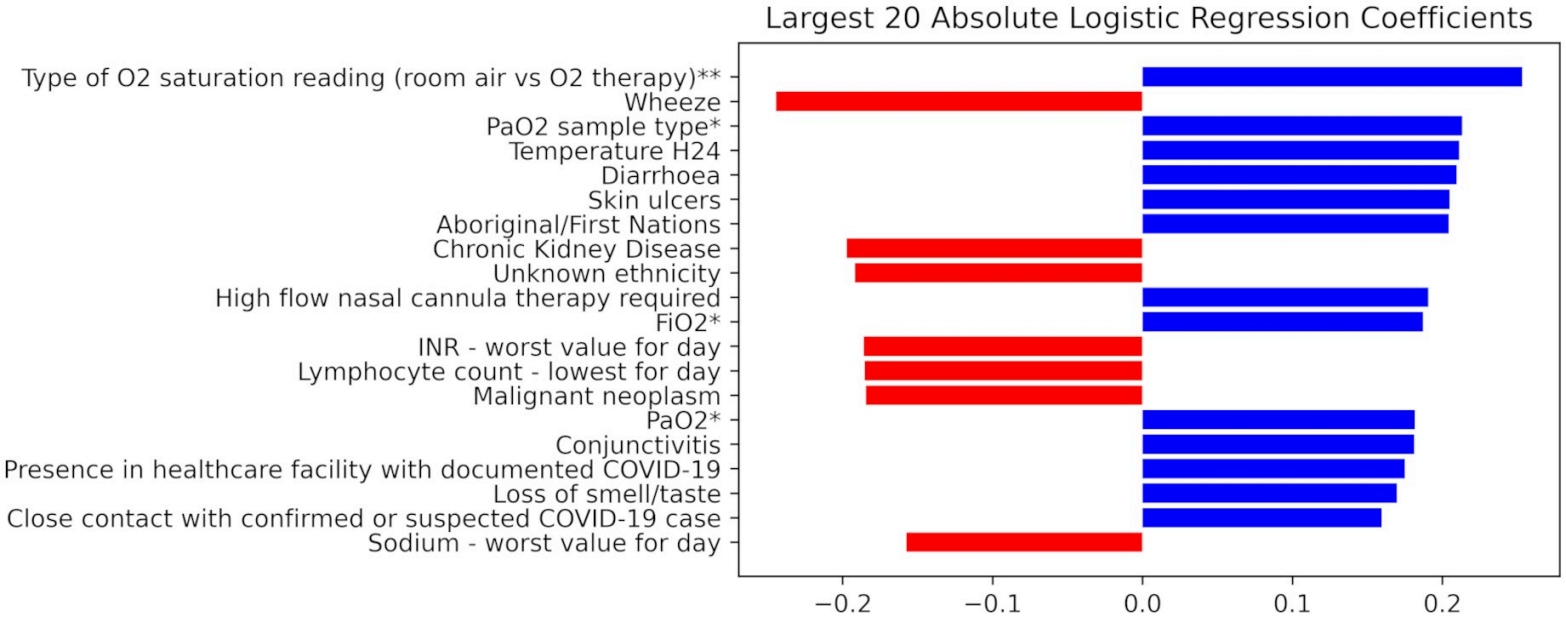
Largest twenty coefficients by absolute value as defined by logistic regression. These represent the model’s weighting of the variable of interest on the outcome (whether or not invasive ventilation will be required by day 2 or 3 of ICU admission). Positive values signify that as variable increases, the risk of requiring invasive ventilation increases. Negative values signify that as the variable decreases, the risk of requiring invasive ventilation decreases. H24—Highest in first 24 hours of hospital admission. ‘Day’ refers to the first 24 hours of ICU admission unless otherwise specified. ’Worst’ refers to the worst value as relating to the APACHE II score. **Closest to pre-intubation on day one. *From ABG with worse P:F (PaO2 divided by FiO2) ratio for the day.

The logistic regression coefficients are shown in Fig 3, with blue and red bar colours representing direct and indirect correlation respectively to the requirement for short term ventilation. There were multiple prominent inputs from a linear standpoint that were not deemed important to random forest classifier or gradient boosted machine. These were an array of both clinical (chronic kidney disease, wheeze, skin ulcers, diarrhoea) and demographic features (Aboriginal ethnicity, presence in a healthcare facility with documented COVID-19, close contact with confirmed or suspected COVID-19 case). That being said, a handful of inputs were deemed to be of high utility in both linear and non-linear modelling, particularly arterial blood gas derived values such arterial partial pressure of oxygen.

Whilst univariate analysis (see Tables 1 and 2) was not used for input filtration, the categorical (high flow nasal cannula therapy) and continuous (pH) inputs deemed to be of greatest significance by univariate analysis nonetheless featured as highly weighted inputs for all three of gradient boosted machine, random forest classifier and logistic regression.

Finally, neither age nor sex featured in the top 20 impactful features of the most performant algorithms in this investigation.

## Discussion

This is the first study to leverage Artificial Intelligence/Machine Learning to identify readily available clinical risk factors for mechanical ventilation in COVID-19 patients admitted to ICU using Australian data [21]. The population in this study represent a ‘grey-area’ cohort who have been deemed unwell enough for ICU admission, however, did not require invasive ventilator support on admission to ICU. The high sensitivity (81%) AI-driven tools developed in this investigation, empower institutions to predict resource allocation for COVID-19 patients at risk of requiring intubation in the short term.

Consensus guidelines on when to intubate patients with severe COVID-19 are lacking and the decision to intubate at present is based on the discretion of the treating physician. Early in the pandemic The Chinese Society of Anaesthesiology Task Force on Airway Management advocated for early intubation of patients showing no improvement in respiratory distress and poor oxygenation (PaO2:FiO2 ratio <150 mmHg) after two hours of high flow oxygen or non-invasive ventilation [22]. Concerns regarding aerosolizing the virus with high-flow oxygen and non-invasive ventilation with subsequent increased risk to healthcare workers, further reinforced calls to intubate early [5]. More recently there has been a shift away from protocolised early intubation. A French prospective multicentre observational study of 245 patients with severe COVID-19 categorised early intubation as within the first two days of ICU admission [23]. Patients in the early intubation cohort had higher rates of pneumonia and bacteraemia, longer lengths of ICU stay and increased 60-day mortality (weighted hazard ratio 1.784, 95% CI 1.07–2.83) [23]. A systematic review of 12 studies involving 8944 critically ill patients with COVID-19 found that timing of intubation had no effect on morbidity or mortality [24]. In the absence of traditional evidence-based guidelines to guide timing of intubation, machine learning algorithms have been proposed as a tool to inform this important clinical decision [11–13].

Utilising a supervised machine learning algorithm, Arvind et al. used 24-hour admission data to predict mechanical ventilation at 72 hours in 4,087 patients admitted to hospital in New York City (United States) with suspected or confirmed COVID-19 [13]. Using a random forest classifier they demonstrated a superior AUC of 0.84 [11]. In a retrospective study of 1,980 COVID-19 patients in Michigan (United States), Yu et al. used a XGBoost machine learning model to predict mechanical ventilation from emergency department data with a prediction accuracy of 86% (96%CI 0.03) and an AUC of 0.68 [12]. In a single centre prospective observational study of 198 patients admitted to an Infectious Disease Clinic in Modena (Italy), Ferrari et al applied GBM machine learning to predict mechanical ventilation with a superior AUC 0f 0.84 [25]. Finally, Heldt et al. applied machine learning to inpatient data of 879 confirmed COVID-19 patients in London (United Kingdom) to predict risk of ICU admission, need for mechanical ventilation and death [11]. Prediction performance was best with random forest and XGBoost models with AUC of 0.87. The algorithms developed in this study are the first to use Australian data to predict outcomes in critically ill patients with COVID-19. The performance of our GBM model with an AUC of 0.68 and sensitivity of 0.81 is inferior to what has been reported internationally [11–13, 25]. This is not surprising; our population were critically ill patients that had already deteriorated to the point of requiring admission to the intensive care unit as opposed to previous machine learning models which had been developed on patients in the emergency department or hospital ward [11–13]. By virtue of a greater severity of illness at baseline we hypothesize that any signals for deterioration to requiring mechanical ventilation will be more dilute in our critically ill cohort.

Strengths of our study include that it was performed using readily available data from a national database in which data collection was performed by experienced research staff using a standardised case report form. The follow-up rate was high with complete data for the primary outcome of invasive ventilation. Our study also represents a unique high acuity cohort for AI modelling of mechanical ventilation risk. Whereas previous studies modelled data from COVID-19 patients in the emergency department [12] and/or hospital ward [11, 13] our cohort were exclusively patients admitted to intensive care. Additionally, it has been shown that the interpretability of the results for time-constrained decision-makers are critical success factors when attempting to integrate automated processes into clinical tasks [26]. Advances in explanatory modelling systems, such as Shapley additive values [20] utilised in this investigation, increase ‘black box’ transparency and thus clinical interpretability. Taken together, these models highlight the potential for artificial intelligence/machine learning to guide clinical decision making across an array of hospital settings.

There are, however, important limitations. Firstly, we restricted our prediction window to a 72-hour interval as per Arvind et al [13]. This meant that a proportion of patients in our cohort who eventually required invasive ventilation beyond day three of their ICU admission were not detected by the model (26/87 30.2%). This shortened forecasting was deemed appropriate given the median time to ventilation was two days (IQR 2–4 days) and events beyond three days were thought to have less mechanistic link to variables collected on the day of admission [13]. Nevertheless, these models do not predict the risk of mechanical ventilation throughout the entire ICU admission. Secondly, increasing the complexity of ML models, especially in the context of smaller sized datasets such as that utilised in this investigation, can cause overfitting [27]. Although our investigation attempted to address the issues of overfitting via active and appropriate choice of pre-training, hyper-parameter selection, and regularisation [28], all models evaluated in the study suffered from overfitting as indicated by performance discrepancies in the training and test sets during cross-validation (see Appendix A of the S1 File). Cross validation during hyperparameter optimisation was also not nested, potentially posing a source of bias [29]. These limitations may impact the external validity of these models. Additionally, despite a rigorous and highly protocolised data collection process, the degree of missingness was high for a selection of the variables. The clinical design of this investigation, however, ensured that these values were missing at random, justifying the implementation of conventional imputation. We tested six of the most commonly used classes of machine learning algorithms which were chosen based on their clinical utility in predicting patient deterioration in critical care settings [11–13, 25]. We acknowledge that there are a multitude of high performing machine learning algorithms with clinical and medical informatics utility and cannot exclude that these additional classes would have superior predictive ability [30–32]. During the capture period Australia experienced two distinct ‘waves’; an initial wave from 27 February to 30 June 2020 and a second wave from 1 July to the 7^th^ of March 2021. Due to insufficient sample size we were unable to undertake a time period analysis by COVID wave. Furthermore, we were not able to compare the model to the current standard being intensivist prediction of mechanical ventilation. Machine learning models may be better, worse or the same as the experienced clinician gestalt.

## Conclusions

ML models based on readily available demographic, observational and laboratory data can reliably predict short term requirements for invasive ventilation in Australian patients with COVID-19 patients not intubated on day one of their ICU admission.

## Supporting information

S1 File (DOCX)

S2 File (ZIP)
