# Supplementary material for: Machine learning predicts the short-term requirement for invasive ventilation among Australian critically ill COVID-19 patients
Source: PLoS One. 2022 Oct 26;17(10):e0276509. doi: 10.1371/journal.pone.0276509 (PMC9604987; doi:10.1371/journal.pone.0276509)
Supplement: S1 File — (DOCX) [file pone.0276509.s001.docx]

**SUPPLEMENTARY MATERIAL (S1)**

Appendix A – Hyperparameters tested, and training metrics

Appendix B – DeLong’s test for classifiers evaluated in this investigation

|  | **Comparison to GBM** | | **Comparison to RF** | |
| --- | --- | --- | --- | --- |
| **Classifier** | ***Z-score*** | ***p-value*** | ***Z-score*** | ***p-value*** |
| GBM | N/A | N/A | 0.82 | 0.41 |
| LR | 2.41 | 0.02 | 3.06 | 2.00E-03 |
| KNN | 2.21 | 0.03 | 2.92 | 0.003 |
| SVM | 3.92 | 8.79E-05 | 4.50 | 6.90E-06 |
| DT | 3.01 | 2.63E-03 | 3.53 | 4.13E-04 |
| RF | -0.82 | 0.41 | N/A | N/A |

*Appendix C – Degree of missingness for input variables*

| **Input variable** | **Degree of missingness (%)** |
| --- | --- |
| History of travel to area with documented COVID cases | 4.3 |
| Close contact with suspected or confirmed COVID-19 case | 13.7 |
| Presence in healthcare facility with documented COVID-19 | 7.3 |
| Presence in laboratory handling COVID-19 samples | 5.0 |
| Arab | 0.0 |
| Black | 0.0 |
| East Asian | 0.0 |
| South Asian | 0.0 |
| West Asian | 0.0 |
| Latin American | 0.0 |
| Caucasian | 0.0 |
| Aboriginal/First Nations | 0.0 |
| Other Ethnicity | 0.0 |
| Unknown Ethnicity | 0.0 |
| Sex at birth | 0.0 |
| Transfer from other health facility | 2.3 |
| History of fever | 2.3 |
| Cough | 2.3 |
| Cough with sputum production | 2.7 |
| Haemoptysis | 2.7 |
| Sore throat | 2.3 |
| Rhinorrhoea | 2.3 |
| Ear pain | 2.7 |
| Wheeze | 2.7 |
| Chest pain | 2.7 |
| Myalgia | 2.7 |
| Joint pain | 2.7 |
| Fatigue | 2.3 |
| Dyspnoea | 2.3 |
| Lower chest wall indrawing | 2.7 |
| Headache | 2.3 |
| Altered conscious state | 2.7 |
| Seizures | 2.7 |
| Abdominal pain | 2.7 |
| Vomiting/Nausea | 2.7 |
| Diarrhoea | 2.7 |
| Conjunctivitis | 2.7 |
| Skin rash | 2.7 |
| Skin ulcers | 2.7 |
| Lymphadenopathy | 2.7 |
| Bleeding(haemorrhage) | 2.7 |
| Bleeding in more than one place | 2.7 |
| Loss of smell/taste | 2.7 |
| Rigors or sweating | 2.3 |
| Severe dehydration | 27.0 |
| Type of oxygen saturation reading closest to pre-intubation on day one | 3.3 |
| Chronic cardiac disease | 2.7 |
| Past ACE inhibitor or A2 blocker use | 4.0 |
| Obesity | 4.0 |
| Chronic pulmonary disease | 2.7 |
| Complicated diabetes | 3.3 |
| Uncomplicated diabetes | 3.7 |
| Asthma | 3.0 |
| Chronic Kidney Disease | 2.7 |
| Rheumatological disorder | 2.7 |
| Moderate or severe liver disease | 2.7 |
| Dementia | 2.7 |
| Mild liver disease | 2.7 |
| Malnutrition | 3.3 |
| Chronic neurological disorder | 2.7 |
| Malignant neoplasm | 2.7 |
| Smoker | 4.3 |
| Chronic haematological disease | 2.7 |
| AIDS/HIV | 3.0 |
| Chronic immunosuppression | 2.7 |
| Readmission | 6.0 |
| PaO2 sample type - from ABG with worst P:F ratio for the day: | 25.3 |
| High flow nasal cannula therapy required | 1.3 |
| Non-invasive ventilation (e.g. BIPAP,CPAP) required | 2.7 |
| Vasopressor support required | 2.7 |
| Prone positioning required | 2.3 |
| Age | 0 |
| Time from onset of symptoms to ICU admission (days) | 2.3 |
| Time spent in hospital prior to ICU admission (hours) | 0.0 |
| Temperature H24 | 2.7 |
| Heart rate H24 | 2.7 |
| Respiratory rate (highest for day) | 2.3 |
| Systolic blood pressure L24 | 2.3 |
| Diastolic blood pressure (from same point as SBP L24) | 2.7 |
| Oxygen saturation (%) closest to pre-intubation on day one | 4.7 |
| Estimated height | 12.7 |
| Estimated weight | 5.3 |
| FiO2 - from ABG with worst P:F ratio for the day | 14.0 |
| SaO2/SpO2 - from ABG with worst P:F ratio for the day | 43.3 |
| PaO2 - from ABG with worst P:F ratio for the day | 26.3 |
| PaCO2 -from ABG with worst P:F ratio for the day | 49.0 |
| pH from ABG with worst P:F for the day | 49.3 |
| HCO3- from from ABG with worst P:F ratio for the day | 49.3 |
| Base excess from ABG with worst P:F ratio for the day | 49.7 |
| GCS - lowest for the day | 35.7 |
| Systolic BP - lowest for the day | 35.7 |
| Diastolic BP (from same time point as lowest SBP on day 1) | 35.7 |
| Mean arterial pressure - lowest for day | 36.0 |
| Daily urine output | 52.7 |
| Platelet count - worst value for day | 50.0 |
| Total bilirubin - worst value for day | 54.0 |
| Lactate - worst value for day | 26.7 |
| Creatinine - worst value for day | 49.7 |
| Number of quadrants in which infiltrates are present on CXR | 67.7 |
| Haemoglobin - lowest value for day | 49.7 |
| WBC count - lowest for day | 25.0 |
| WBC count - highest for day | 59.0 |
| Lymphocyte count - lowest for day | 50.7 |
| Neutrophil count - lowest for day | 50.7 |
| Haematocrit - worst value for day | 51.3 |
| APTT/APTR - worst value for day | 69.0 |
| PT - worst value for day | 76.7 |
| INR - worst value for day | 67.7 |
| ALT/SGPT - worst value for day | 56.0 |
| AST/SGOT - worst value for day | 70.7 |
| Glucose - highest for day | 51.7 |
| Blood Urea Nitrogen - worst value for day | 53.7 |
| Sodium - worst value for day | 49.7 |
| Potassium - worst value for day | 50.0 |
| CRP - worst value for day | 61.3 |
| Daily fluid balance | 63.3 |

*Appendix D – Sensitivity analysis comparing ‘full’ and ‘reduced variable’ versions of each model*

|  | **All variables AUC (Mean, SD)** | | **Reduced variables AUC (Mean, SD)** | | **Significance*** |
| --- | --- | --- | --- | --- | --- |
| KNN | 0.595 | 0.077 | 0.613 | 0.084 | N/A |
| SVC | 0.658 | 0.084 | 0.664 | 0.059 | p = 0.798 |
| DT | 0.512 | 0.088 | 0.522 | 0.085 | p = 0.720 |
| GBM | 0.711 | 0.062 | 0.664 | 0.089 | N/A |
| LR | 0.601 | 0.091 | 0.611 | 0.098 | p = 0.743 |
| RF | 0.702 | 0.053 | 0.689 | 0.068 | N/A |

*Welch two-sample T-test comparing ‘full’ and ‘reduced’ models. Sensitivity analysis was performed only for clarification in cases where there was an apparent advantage yielded by variable reduction.
