# Supplementary figures and images for: Machine learning predicts the short-term requirement for invasive ventilation among Australian critically ill COVID-19 patients (part 2 of 2)
Source: PLoS One. 2022 Oct 26;17(10):e0276509. doi: 10.1371/journal.pone.0276509 (PMC9604987; doi:10.1371/journal.pone.0276509)

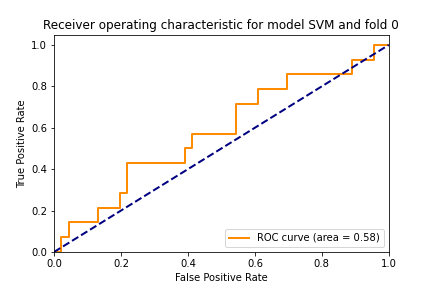

Supplement: S2 File — (ZIP) [file pone.0276509.s002.zip › revised_plots/SVM/SVM_0.png]

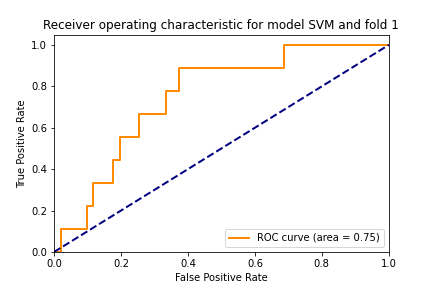

Supplement: S2 File — (ZIP) [file pone.0276509.s002.zip › revised_plots/SVM/SVM_1.png]

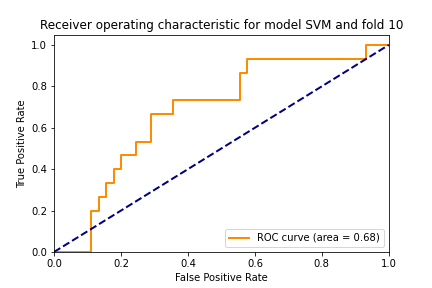

Supplement: S2 File — (ZIP) [file pone.0276509.s002.zip › revised_plots/SVM/SVM_10.png]

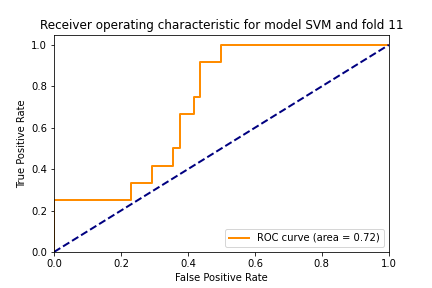

Supplement: S2 File — (ZIP) [file pone.0276509.s002.zip › revised_plots/SVM/SVM_11.png]

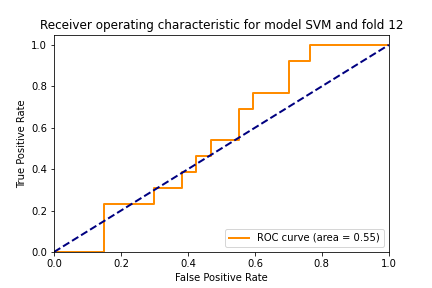

Supplement: S2 File — (ZIP) [file pone.0276509.s002.zip › revised_plots/SVM/SVM_12.png]

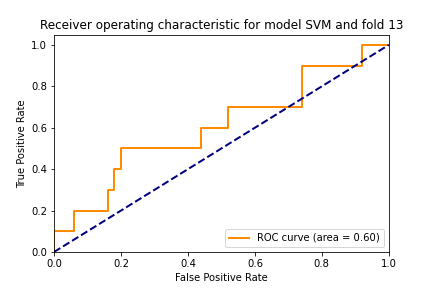

Supplement: S2 File — (ZIP) [file pone.0276509.s002.zip › revised_plots/SVM/SVM_13.png]

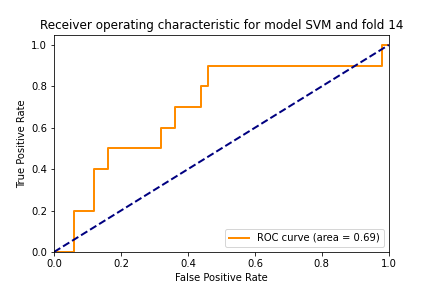

Supplement: S2 File — (ZIP) [file pone.0276509.s002.zip › revised_plots/SVM/SVM_14.png]

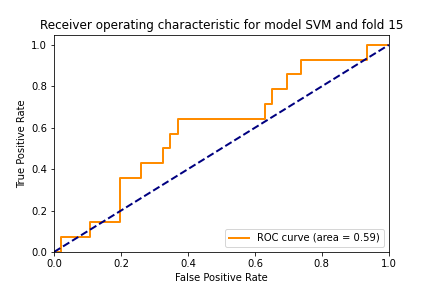

Supplement: S2 File — (ZIP) [file pone.0276509.s002.zip › revised_plots/SVM/SVM_15.png]

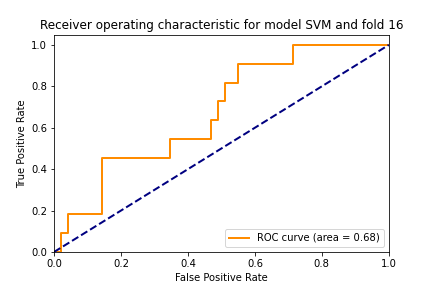

Supplement: S2 File — (ZIP) [file pone.0276509.s002.zip › revised_plots/SVM/SVM_16.png]

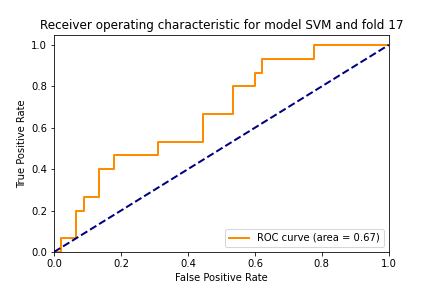

Supplement: S2 File — (ZIP) [file pone.0276509.s002.zip › revised_plots/SVM/SVM_17.png]

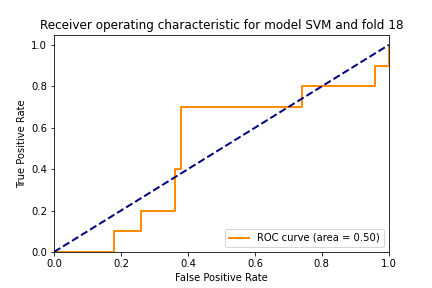

Supplement: S2 File — (ZIP) [file pone.0276509.s002.zip › revised_plots/SVM/SVM_18.png]

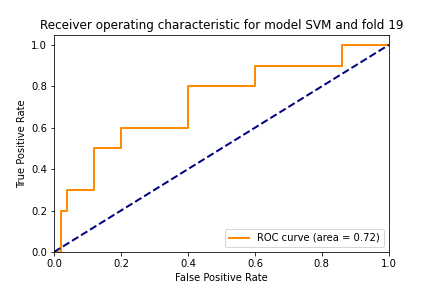

Supplement: S2 File — (ZIP) [file pone.0276509.s002.zip › revised_plots/SVM/SVM_19.png]

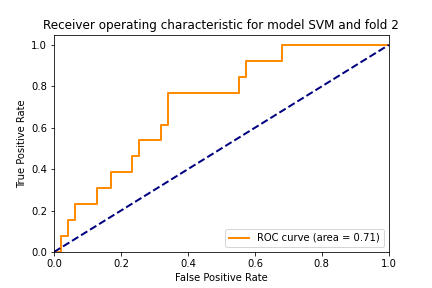

Supplement: S2 File — (ZIP) [file pone.0276509.s002.zip › revised_plots/SVM/SVM_2.png]

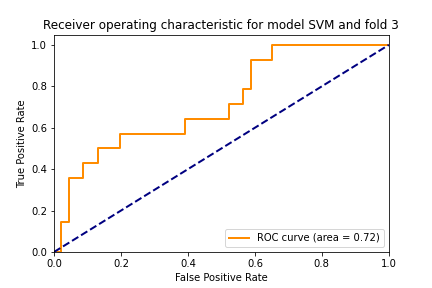

Supplement: S2 File — (ZIP) [file pone.0276509.s002.zip › revised_plots/SVM/SVM_3.png]

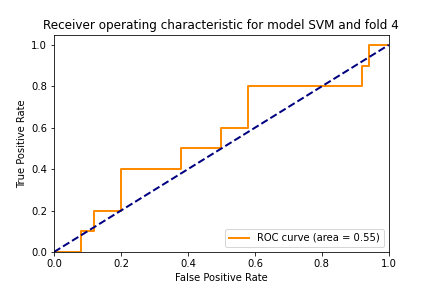

Supplement: S2 File — (ZIP) [file pone.0276509.s002.zip › revised_plots/SVM/SVM_4.png]

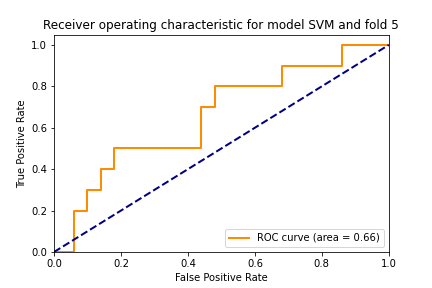

Supplement: S2 File — (ZIP) [file pone.0276509.s002.zip › revised_plots/SVM/SVM_5.png]

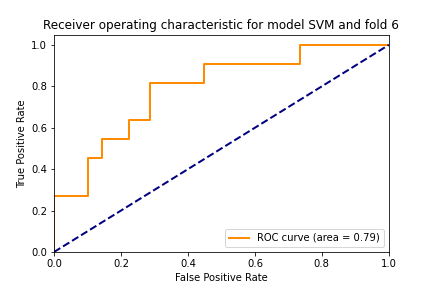

Supplement: S2 File — (ZIP) [file pone.0276509.s002.zip › revised_plots/SVM/SVM_6.png]

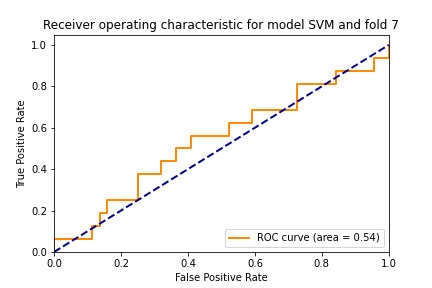

Supplement: S2 File — (ZIP) [file pone.0276509.s002.zip › revised_plots/SVM/SVM_7.png]

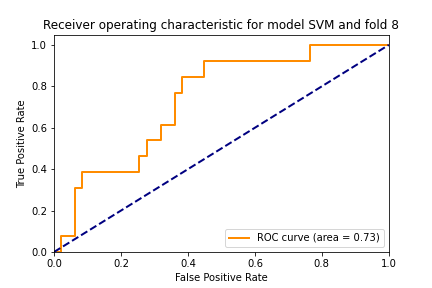

Supplement: S2 File — (ZIP) [file pone.0276509.s002.zip › revised_plots/SVM/SVM_8.png]

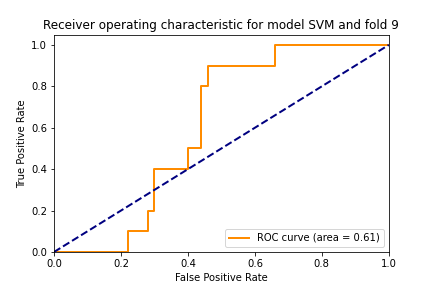

Supplement: S2 File — (ZIP) [file pone.0276509.s002.zip › revised_plots/SVM/SVM_9.png]
