# Supplementary figures and images for: Machine learning predicts the short-term requirement for invasive ventilation among Australian critically ill COVID-19 patients (part 1 of 2)
Source: PLoS One. 2022 Oct 26;17(10):e0276509. doi: 10.1371/journal.pone.0276509 (PMC9604987; doi:10.1371/journal.pone.0276509)

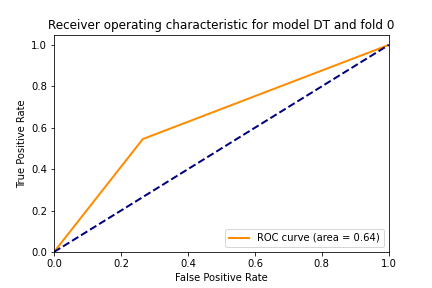

Supplement: S2 File — (ZIP) [file pone.0276509.s002.zip › revised_plots/DT/DT_0.png]

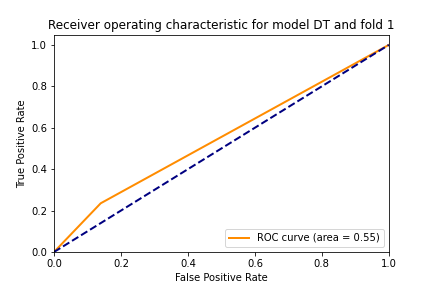

Supplement: S2 File — (ZIP) [file pone.0276509.s002.zip › revised_plots/DT/DT_1.png]

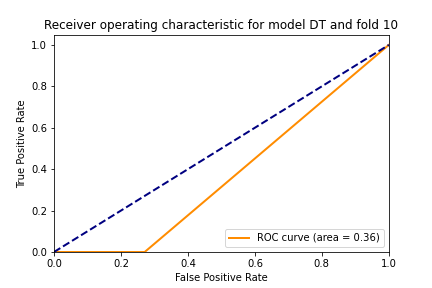

Supplement: S2 File — (ZIP) [file pone.0276509.s002.zip › revised_plots/DT/DT_10.png]

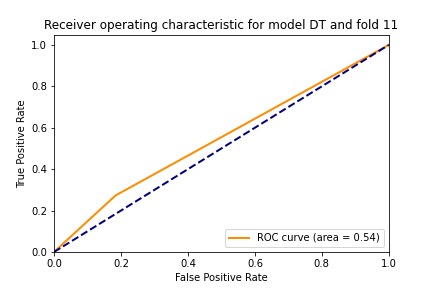

Supplement: S2 File — (ZIP) [file pone.0276509.s002.zip › revised_plots/DT/DT_11.png]

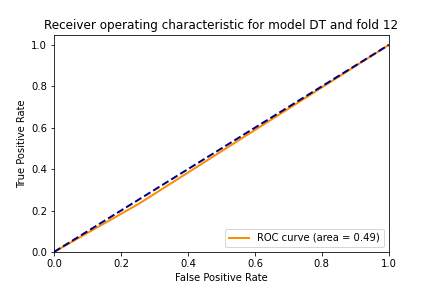

Supplement: S2 File — (ZIP) [file pone.0276509.s002.zip › revised_plots/DT/DT_12.png]

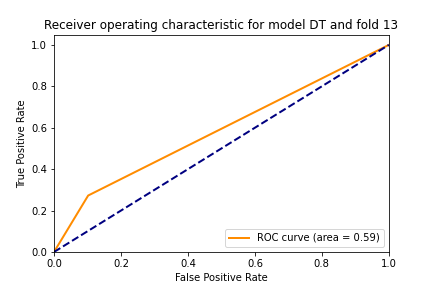

Supplement: S2 File — (ZIP) [file pone.0276509.s002.zip › revised_plots/DT/DT_13.png]

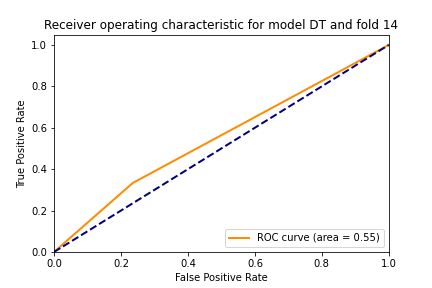

Supplement: S2 File — (ZIP) [file pone.0276509.s002.zip › revised_plots/DT/DT_14.png]

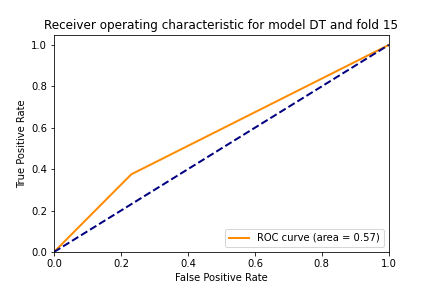

Supplement: S2 File — (ZIP) [file pone.0276509.s002.zip › revised_plots/DT/DT_15.png]

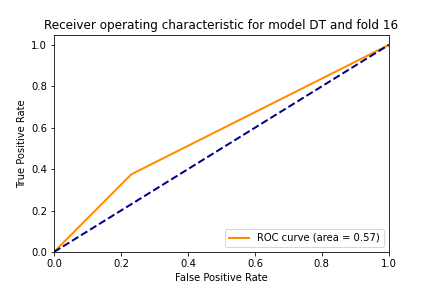

Supplement: S2 File — (ZIP) [file pone.0276509.s002.zip › revised_plots/DT/DT_16.png]

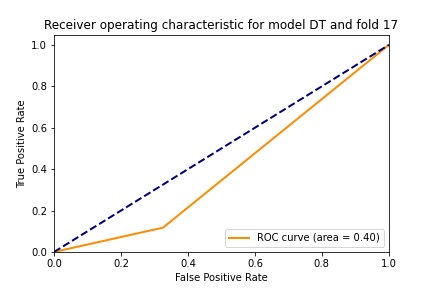

Supplement: S2 File — (ZIP) [file pone.0276509.s002.zip › revised_plots/DT/DT_17.png]

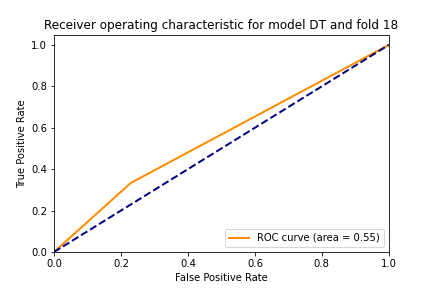

Supplement: S2 File — (ZIP) [file pone.0276509.s002.zip › revised_plots/DT/DT_18.png]

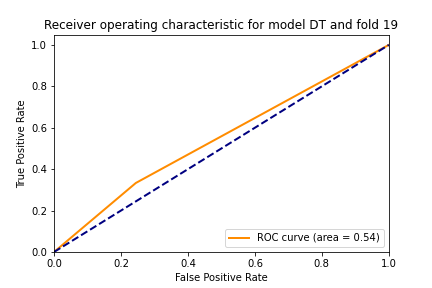

Supplement: S2 File — (ZIP) [file pone.0276509.s002.zip › revised_plots/DT/DT_19.png]

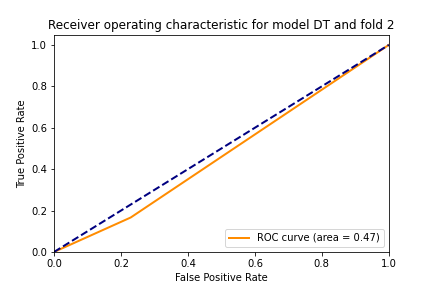

Supplement: S2 File — (ZIP) [file pone.0276509.s002.zip › revised_plots/DT/DT_2.png]

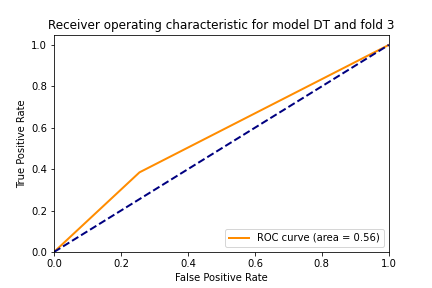

Supplement: S2 File — (ZIP) [file pone.0276509.s002.zip › revised_plots/DT/DT_3.png]

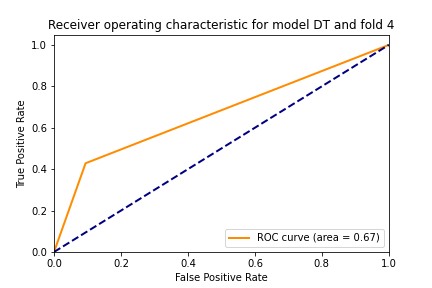

Supplement: S2 File — (ZIP) [file pone.0276509.s002.zip › revised_plots/DT/DT_4.png]

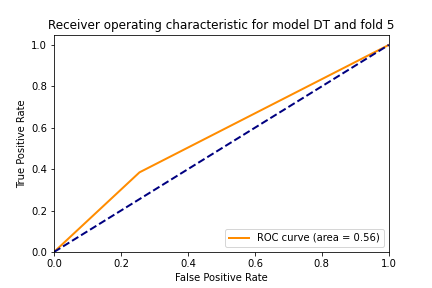

Supplement: S2 File — (ZIP) [file pone.0276509.s002.zip › revised_plots/DT/DT_5.png]

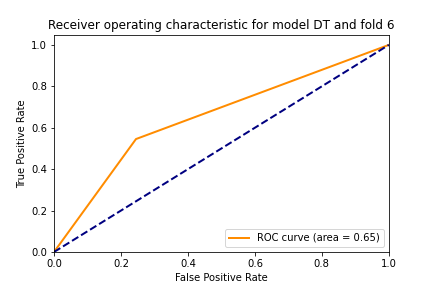

Supplement: S2 File — (ZIP) [file pone.0276509.s002.zip › revised_plots/DT/DT_6.png]

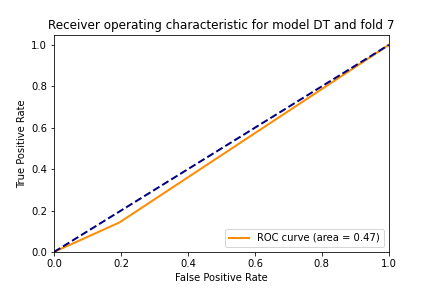

Supplement: S2 File — (ZIP) [file pone.0276509.s002.zip › revised_plots/DT/DT_7.png]

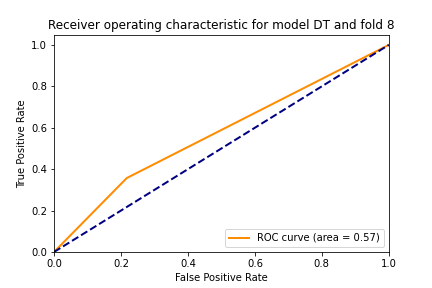

Supplement: S2 File — (ZIP) [file pone.0276509.s002.zip › revised_plots/DT/DT_8.png]

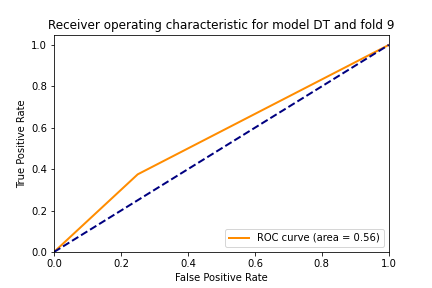

Supplement: S2 File — (ZIP) [file pone.0276509.s002.zip › revised_plots/DT/DT_9.png]

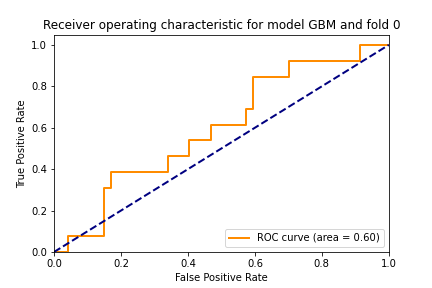

Supplement: S2 File — (ZIP) [file pone.0276509.s002.zip › revised_plots/GBM/GBM_0.png]

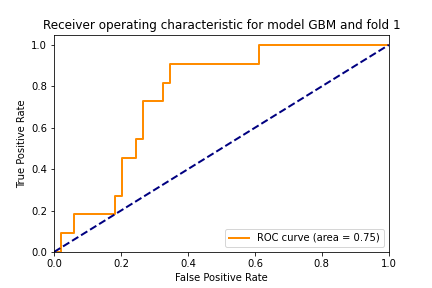

Supplement: S2 File — (ZIP) [file pone.0276509.s002.zip › revised_plots/GBM/GBM_1.png]

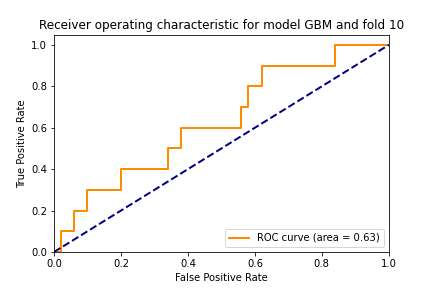

Supplement: S2 File — (ZIP) [file pone.0276509.s002.zip › revised_plots/GBM/GBM_10.png]

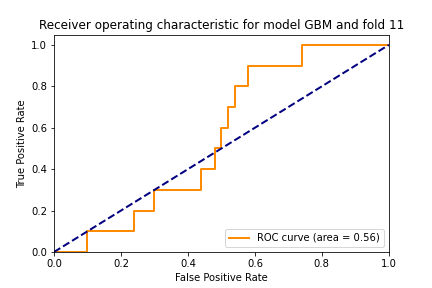

Supplement: S2 File — (ZIP) [file pone.0276509.s002.zip › revised_plots/GBM/GBM_11.png]

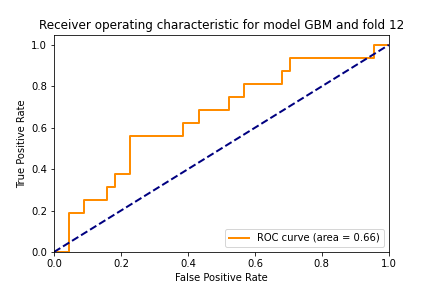

Supplement: S2 File — (ZIP) [file pone.0276509.s002.zip › revised_plots/GBM/GBM_12.png]

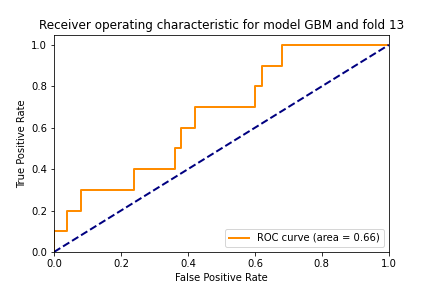

Supplement: S2 File — (ZIP) [file pone.0276509.s002.zip › revised_plots/GBM/GBM_13.png]

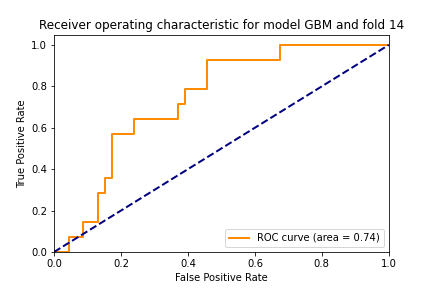

Supplement: S2 File — (ZIP) [file pone.0276509.s002.zip › revised_plots/GBM/GBM_14.png]

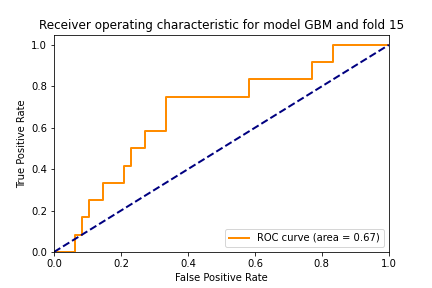

Supplement: S2 File — (ZIP) [file pone.0276509.s002.zip › revised_plots/GBM/GBM_15.png]

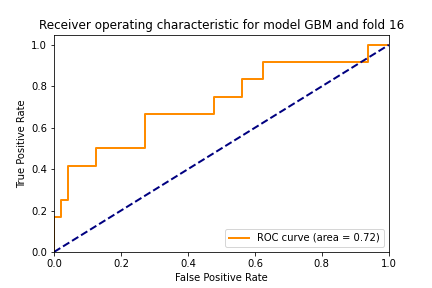

Supplement: S2 File — (ZIP) [file pone.0276509.s002.zip › revised_plots/GBM/GBM_16.png]

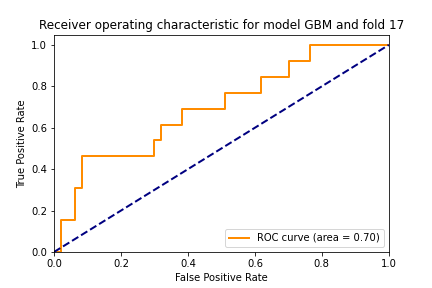

Supplement: S2 File — (ZIP) [file pone.0276509.s002.zip › revised_plots/GBM/GBM_17.png]

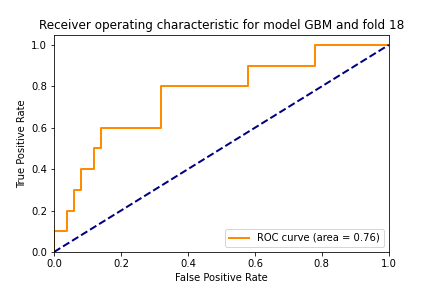

Supplement: S2 File — (ZIP) [file pone.0276509.s002.zip › revised_plots/GBM/GBM_18.png]

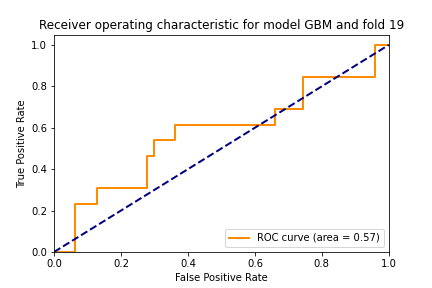

Supplement: S2 File — (ZIP) [file pone.0276509.s002.zip › revised_plots/GBM/GBM_19.png]

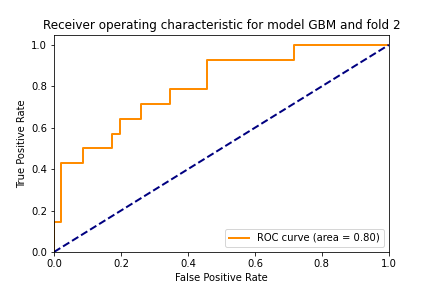

Supplement: S2 File — (ZIP) [file pone.0276509.s002.zip › revised_plots/GBM/GBM_2.png]

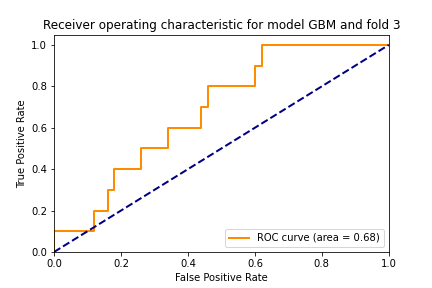

Supplement: S2 File — (ZIP) [file pone.0276509.s002.zip › revised_plots/GBM/GBM_3.png]

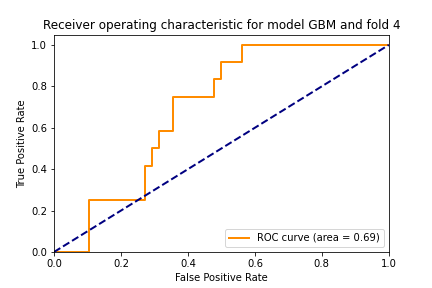

Supplement: S2 File — (ZIP) [file pone.0276509.s002.zip › revised_plots/GBM/GBM_4.png]

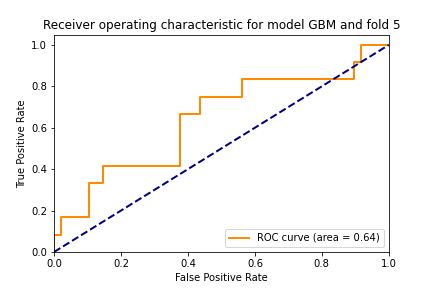

Supplement: S2 File — (ZIP) [file pone.0276509.s002.zip › revised_plots/GBM/GBM_5.png]

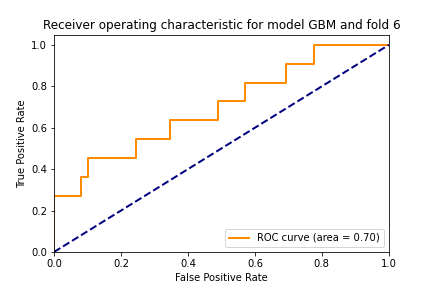

Supplement: S2 File — (ZIP) [file pone.0276509.s002.zip › revised_plots/GBM/GBM_6.png]

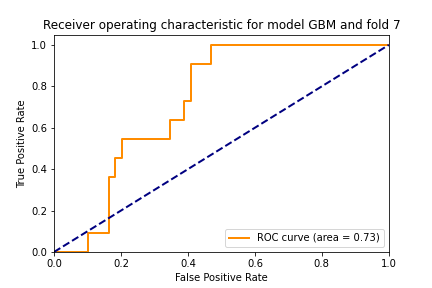

Supplement: S2 File — (ZIP) [file pone.0276509.s002.zip › revised_plots/GBM/GBM_7.png]

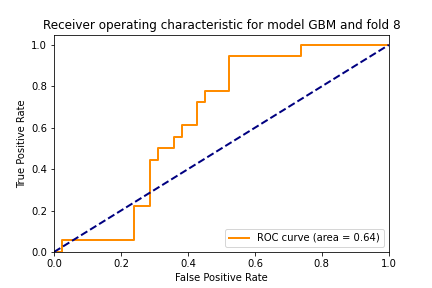

Supplement: S2 File — (ZIP) [file pone.0276509.s002.zip › revised_plots/GBM/GBM_8.png]

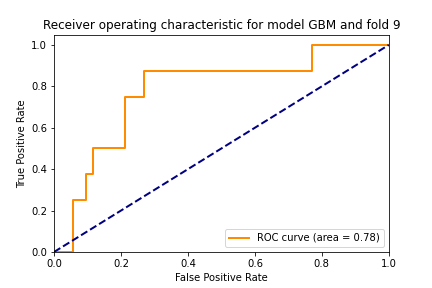

Supplement: S2 File — (ZIP) [file pone.0276509.s002.zip › revised_plots/GBM/GBM_9.png]

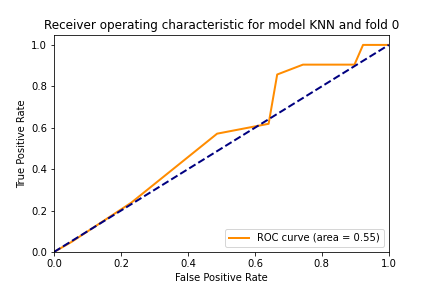

Supplement: S2 File — (ZIP) [file pone.0276509.s002.zip › revised_plots/KNN/KNN_0.png]

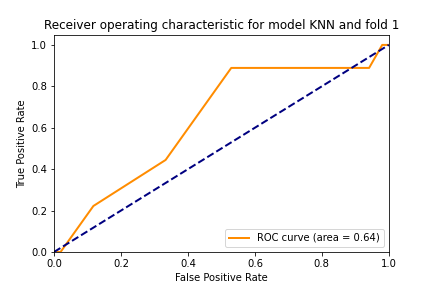

Supplement: S2 File — (ZIP) [file pone.0276509.s002.zip › revised_plots/KNN/KNN_1.png]

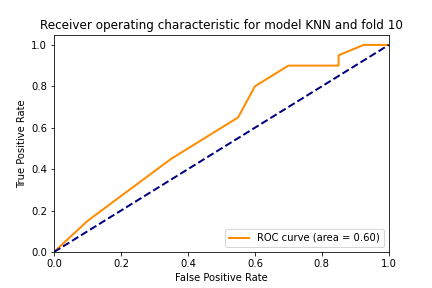

Supplement: S2 File — (ZIP) [file pone.0276509.s002.zip › revised_plots/KNN/KNN_10.png]

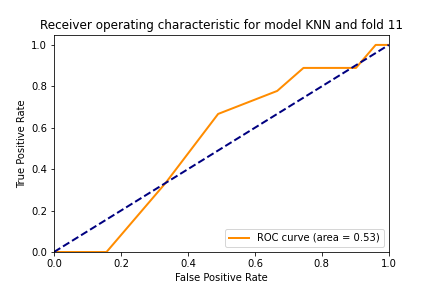

Supplement: S2 File — (ZIP) [file pone.0276509.s002.zip › revised_plots/KNN/KNN_11.png]

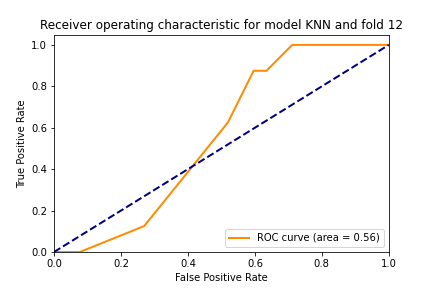

Supplement: S2 File — (ZIP) [file pone.0276509.s002.zip › revised_plots/KNN/KNN_12.png]

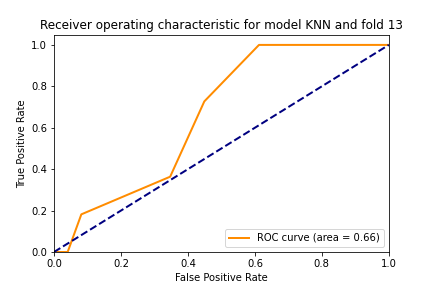

Supplement: S2 File — (ZIP) [file pone.0276509.s002.zip › revised_plots/KNN/KNN_13.png]

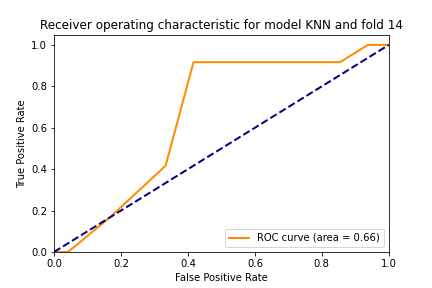

Supplement: S2 File — (ZIP) [file pone.0276509.s002.zip › revised_plots/KNN/KNN_14.png]

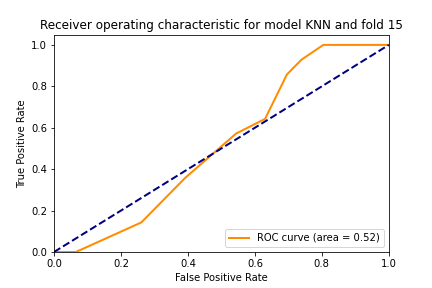

Supplement: S2 File — (ZIP) [file pone.0276509.s002.zip › revised_plots/KNN/KNN_15.png]

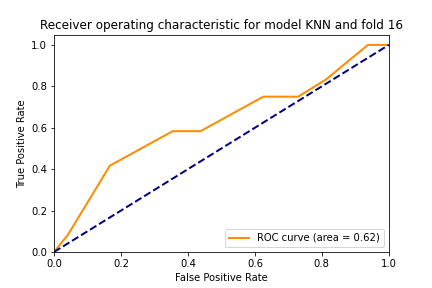

Supplement: S2 File — (ZIP) [file pone.0276509.s002.zip › revised_plots/KNN/KNN_16.png]

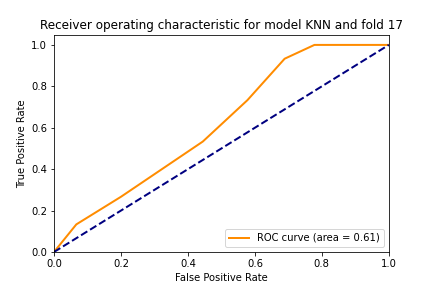

Supplement: S2 File — (ZIP) [file pone.0276509.s002.zip › revised_plots/KNN/KNN_17.png]

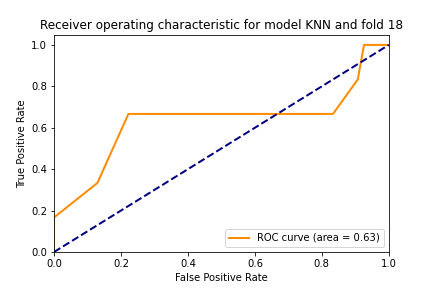

Supplement: S2 File — (ZIP) [file pone.0276509.s002.zip › revised_plots/KNN/KNN_18.png]

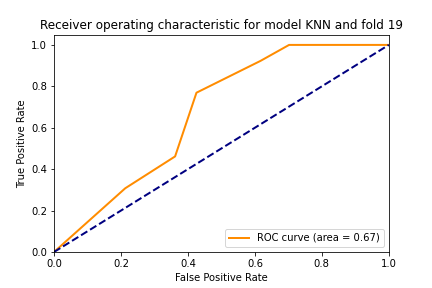

Supplement: S2 File — (ZIP) [file pone.0276509.s002.zip › revised_plots/KNN/KNN_19.png]

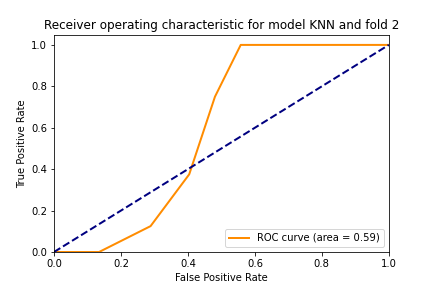

Supplement: S2 File — (ZIP) [file pone.0276509.s002.zip › revised_plots/KNN/KNN_2.png]

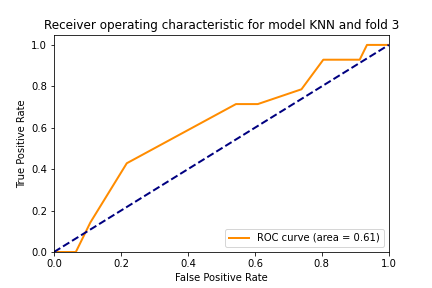

Supplement: S2 File — (ZIP) [file pone.0276509.s002.zip › revised_plots/KNN/KNN_3.png]

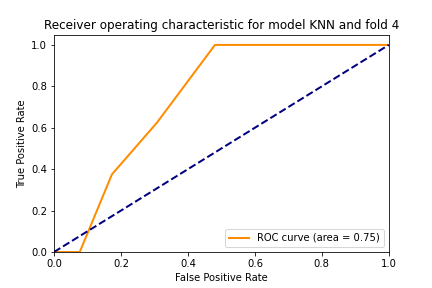

Supplement: S2 File — (ZIP) [file pone.0276509.s002.zip › revised_plots/KNN/KNN_4.png]

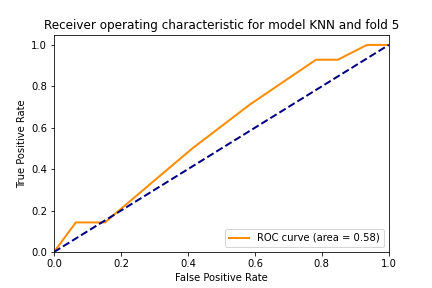

Supplement: S2 File — (ZIP) [file pone.0276509.s002.zip › revised_plots/KNN/KNN_5.png]

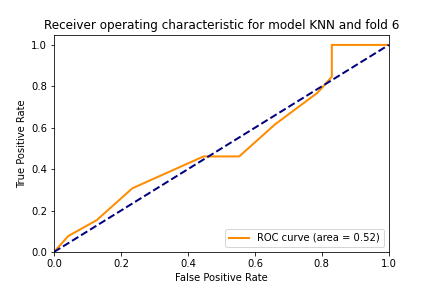

Supplement: S2 File — (ZIP) [file pone.0276509.s002.zip › revised_plots/KNN/KNN_6.png]

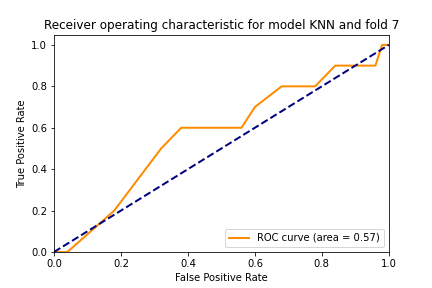

Supplement: S2 File — (ZIP) [file pone.0276509.s002.zip › revised_plots/KNN/KNN_7.png]

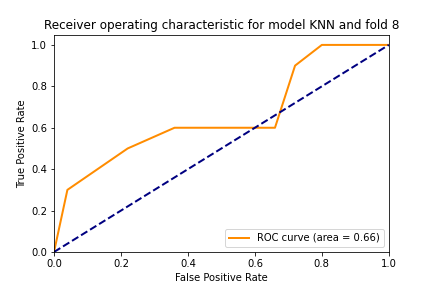

Supplement: S2 File — (ZIP) [file pone.0276509.s002.zip › revised_plots/KNN/KNN_8.png]

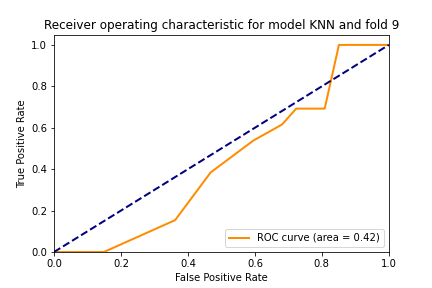

Supplement: S2 File — (ZIP) [file pone.0276509.s002.zip › revised_plots/KNN/KNN_9.png]

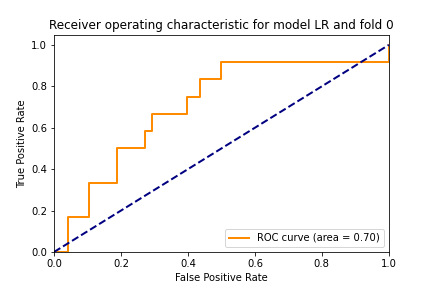

Supplement: S2 File — (ZIP) [file pone.0276509.s002.zip › revised_plots/LR/LR_0.png]

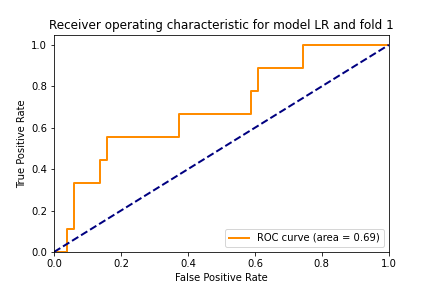

Supplement: S2 File — (ZIP) [file pone.0276509.s002.zip › revised_plots/LR/LR_1.png]

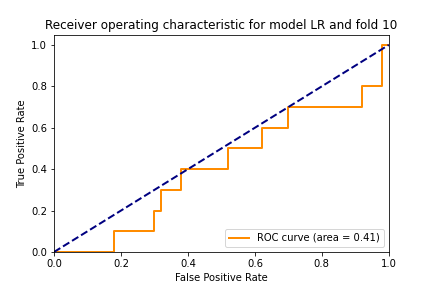

Supplement: S2 File — (ZIP) [file pone.0276509.s002.zip › revised_plots/LR/LR_10.png]

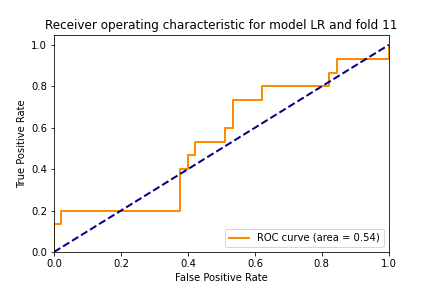

Supplement: S2 File — (ZIP) [file pone.0276509.s002.zip › revised_plots/LR/LR_11.png]

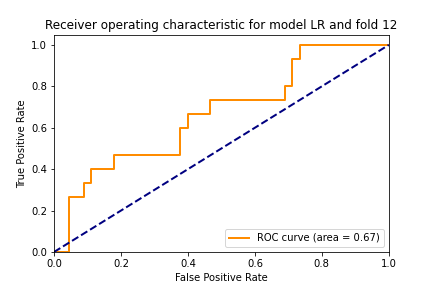

Supplement: S2 File — (ZIP) [file pone.0276509.s002.zip › revised_plots/LR/LR_12.png]

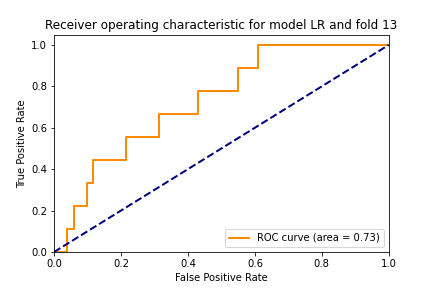

Supplement: S2 File — (ZIP) [file pone.0276509.s002.zip › revised_plots/LR/LR_13.png]

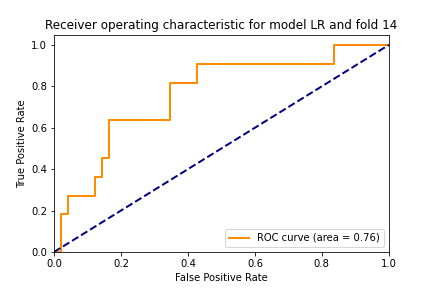

Supplement: S2 File — (ZIP) [file pone.0276509.s002.zip › revised_plots/LR/LR_14.png]

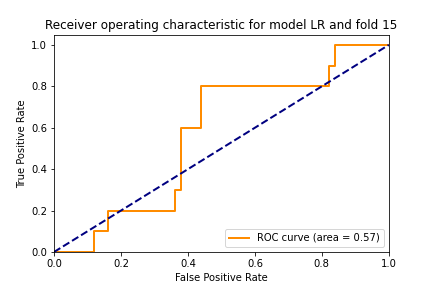

Supplement: S2 File — (ZIP) [file pone.0276509.s002.zip › revised_plots/LR/LR_15.png]

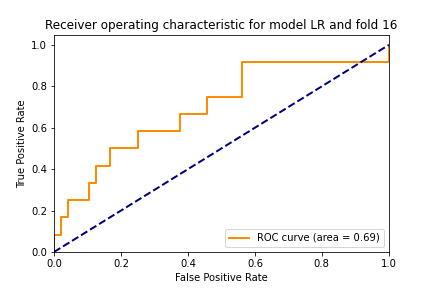

Supplement: S2 File — (ZIP) [file pone.0276509.s002.zip › revised_plots/LR/LR_16.png]

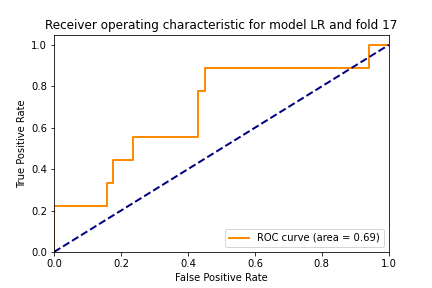

Supplement: S2 File — (ZIP) [file pone.0276509.s002.zip › revised_plots/LR/LR_17.png]

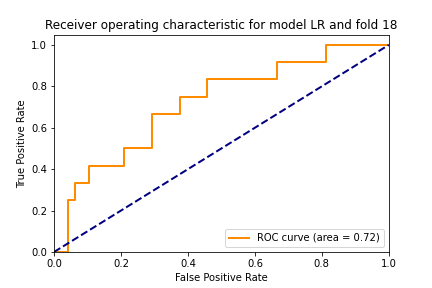

Supplement: S2 File — (ZIP) [file pone.0276509.s002.zip › revised_plots/LR/LR_18.png]

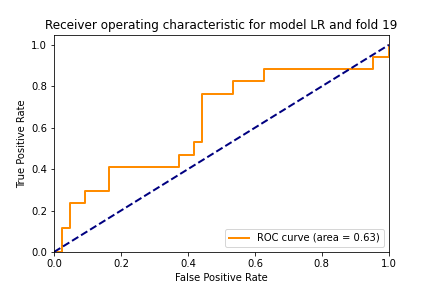

Supplement: S2 File — (ZIP) [file pone.0276509.s002.zip › revised_plots/LR/LR_19.png]

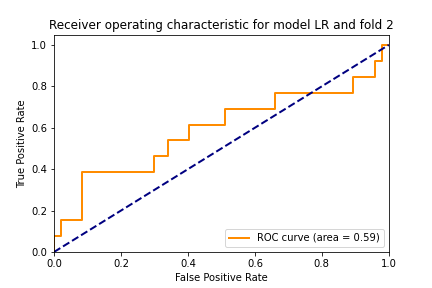

Supplement: S2 File — (ZIP) [file pone.0276509.s002.zip › revised_plots/LR/LR_2.png]

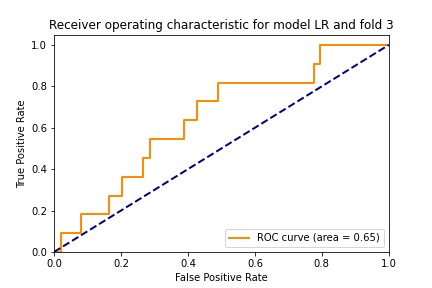

Supplement: S2 File — (ZIP) [file pone.0276509.s002.zip › revised_plots/LR/LR_3.png]

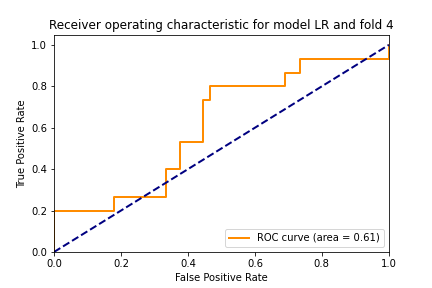

Supplement: S2 File — (ZIP) [file pone.0276509.s002.zip › revised_plots/LR/LR_4.png]

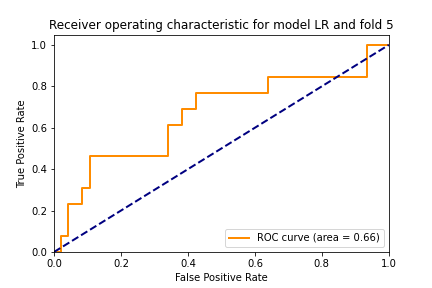

Supplement: S2 File — (ZIP) [file pone.0276509.s002.zip › revised_plots/LR/LR_5.png]

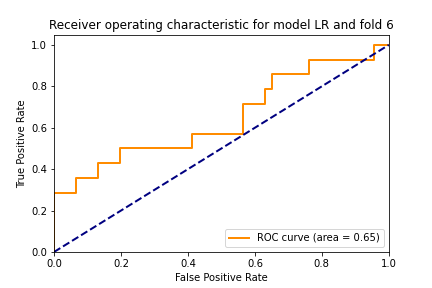

Supplement: S2 File — (ZIP) [file pone.0276509.s002.zip › revised_plots/LR/LR_6.png]

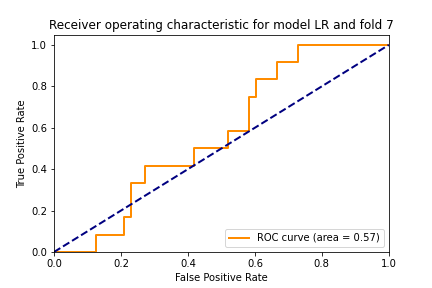

Supplement: S2 File — (ZIP) [file pone.0276509.s002.zip › revised_plots/LR/LR_7.png]

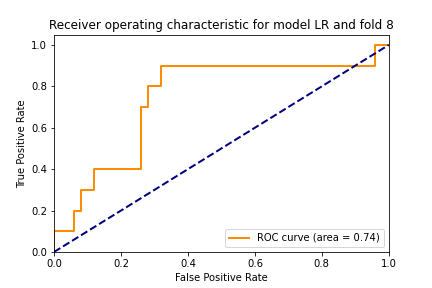

Supplement: S2 File — (ZIP) [file pone.0276509.s002.zip › revised_plots/LR/LR_8.png]

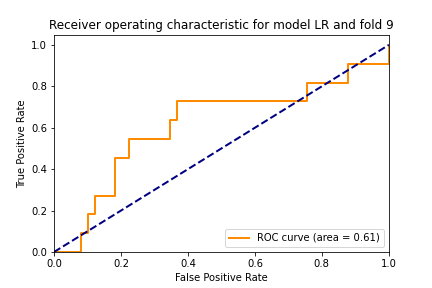

Supplement: S2 File — (ZIP) [file pone.0276509.s002.zip › revised_plots/LR/LR_9.png]

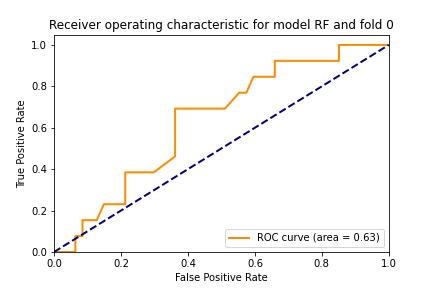

Supplement: S2 File — (ZIP) [file pone.0276509.s002.zip › revised_plots/RF/RF_0.png]

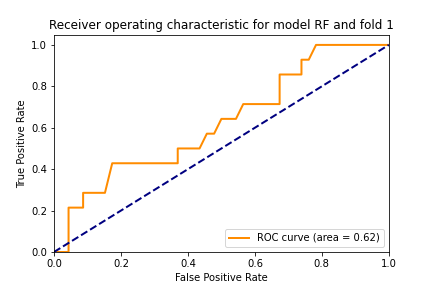

Supplement: S2 File — (ZIP) [file pone.0276509.s002.zip › revised_plots/RF/RF_1.png]

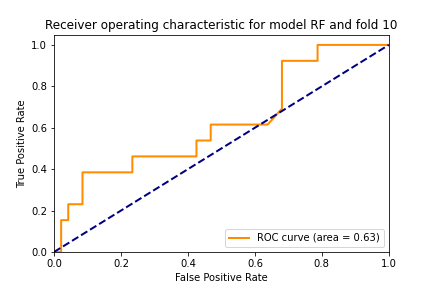

Supplement: S2 File — (ZIP) [file pone.0276509.s002.zip › revised_plots/RF/RF_10.png]

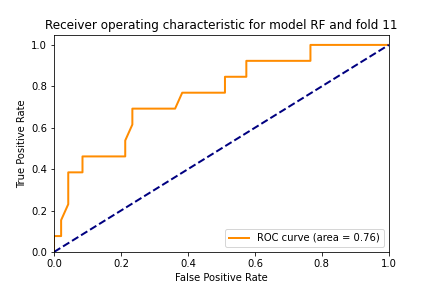

Supplement: S2 File — (ZIP) [file pone.0276509.s002.zip › revised_plots/RF/RF_11.png]

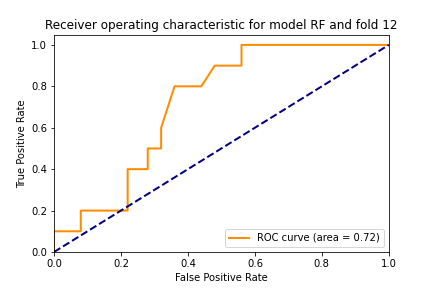

Supplement: S2 File — (ZIP) [file pone.0276509.s002.zip › revised_plots/RF/RF_12.png]

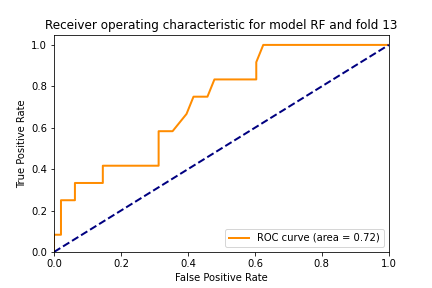

Supplement: S2 File — (ZIP) [file pone.0276509.s002.zip › revised_plots/RF/RF_13.png]

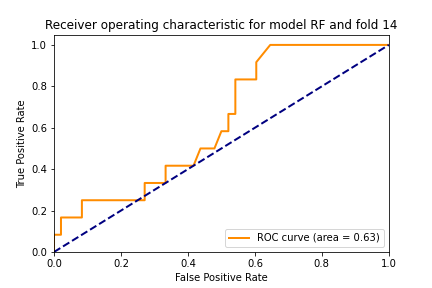

Supplement: S2 File — (ZIP) [file pone.0276509.s002.zip › revised_plots/RF/RF_14.png]

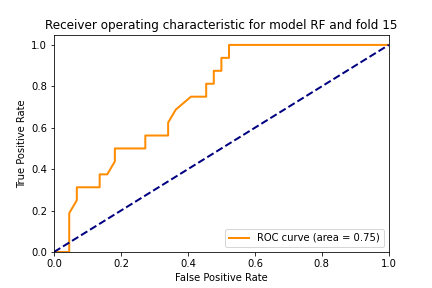

Supplement: S2 File — (ZIP) [file pone.0276509.s002.zip › revised_plots/RF/RF_15.png]

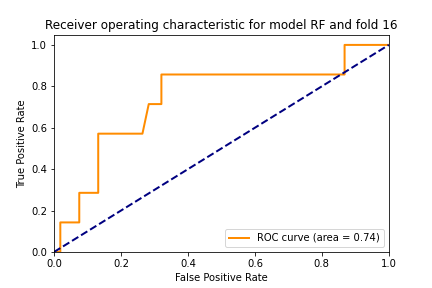

Supplement: S2 File — (ZIP) [file pone.0276509.s002.zip › revised_plots/RF/RF_16.png]

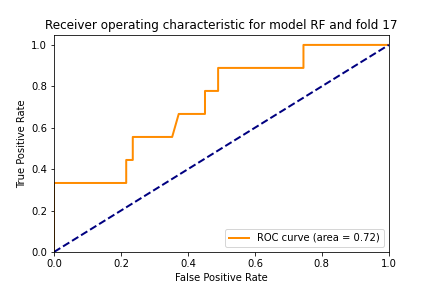

Supplement: S2 File — (ZIP) [file pone.0276509.s002.zip › revised_plots/RF/RF_17.png]

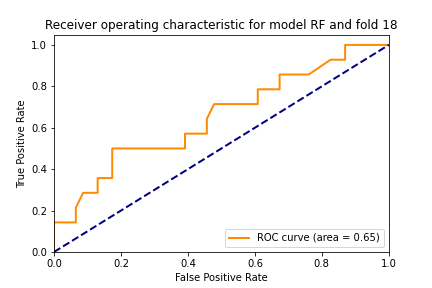

Supplement: S2 File — (ZIP) [file pone.0276509.s002.zip › revised_plots/RF/RF_18.png]

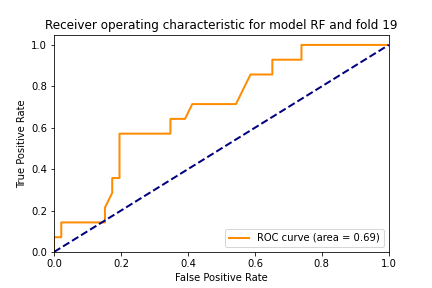

Supplement: S2 File — (ZIP) [file pone.0276509.s002.zip › revised_plots/RF/RF_19.png]

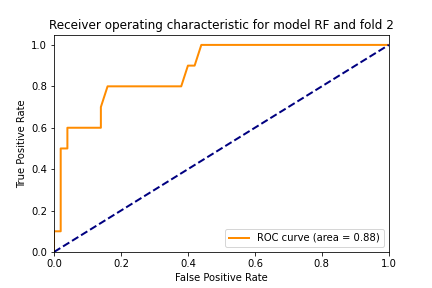

Supplement: S2 File — (ZIP) [file pone.0276509.s002.zip › revised_plots/RF/RF_2.png]

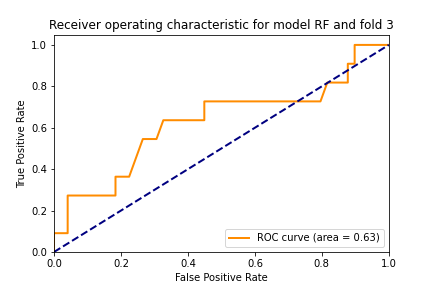

Supplement: S2 File — (ZIP) [file pone.0276509.s002.zip › revised_plots/RF/RF_3.png]

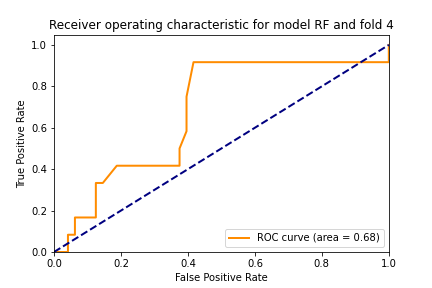

Supplement: S2 File — (ZIP) [file pone.0276509.s002.zip › revised_plots/RF/RF_4.png]

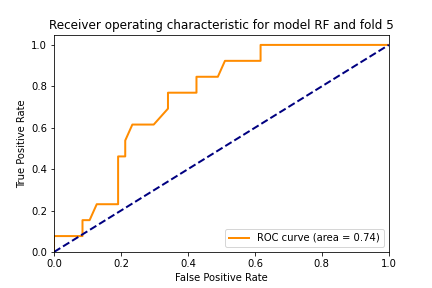

Supplement: S2 File — (ZIP) [file pone.0276509.s002.zip › revised_plots/RF/RF_5.png]

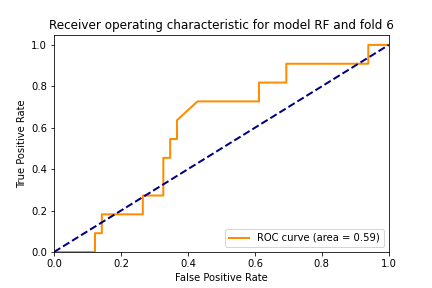

Supplement: S2 File — (ZIP) [file pone.0276509.s002.zip › revised_plots/RF/RF_6.png]

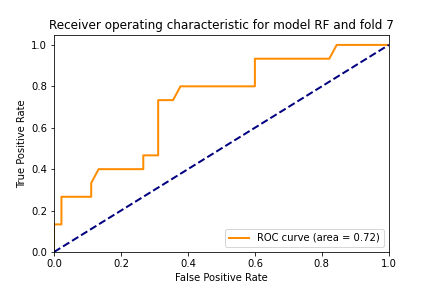

Supplement: S2 File — (ZIP) [file pone.0276509.s002.zip › revised_plots/RF/RF_7.png]

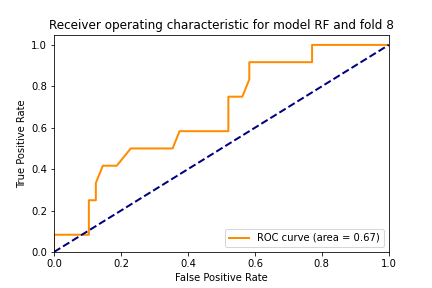

Supplement: S2 File — (ZIP) [file pone.0276509.s002.zip › revised_plots/RF/RF_8.png]

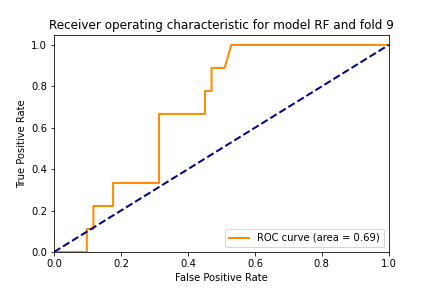

Supplement: S2 File — (ZIP) [file pone.0276509.s002.zip › revised_plots/RF/RF_9.png]
